# Description of a New Eyeless Cavefish Species Using Integrative Taxonomic Methods—*Sinocyclocheilus changlensis* (Cypriniformes, Cyprinidae), From Guangxi, China

**DOI:** 10.1002/ece3.72370

**Published:** 2025-11-19

**Authors:** Yewei Liu, Tingru Mao, Hiranya Sudasinghe, Jiajun Zhou, Rongjiao Chen, Jian Yang, Madhava Meegaskumbura

**Affiliations:** ^1^ Guangxi Key Laboratory for Forest Ecology and Conservation, College of Forestry Guangxi University Nanning Guangxi People's Republic of China; ^2^ Division of Evolutionary Ecology, Institute of Ecology and Evolution University of Bern Bern Switzerland; ^3^ Naturhistorisches Museum Bern Bern Switzerland; ^4^ Zhejiang Forest Resource Monitoring Center Hangzhou Zhejiang People's Republic of China; ^5^ Key Laboratory of Environment Change and Resource Use, Beibu Gulf Nanning Normal University Nanning Guangxi People's Republic of China

**Keywords:** blindness, cavefish, Hongshui River, landmarks, molecular systematics, morphology, *Sinocyclocheilus*, stygomorphic

## Abstract

The extensive limestone landscapes of southwestern China form one of the world's largest karst regions, providing ideal conditions for cavefish evolution. Within this region, *Sinocyclocheilus*, the most speciose cavefish genus globally, comprises 84 species adapted to dark environments. Despite the many species, the region is still poorly explored, with new species currently being added to the total. Here, using integrative taxonomic methods involving morphological and molecular analyses, we describe *Sinocyclocheilus changlensis*, a new troglobitic species discovered in a cave in central Guangxi, China. This species is characterized by the absence of eyes, an unpigmented and complete scaled body, and a forked, horn‐like structure at the dorsal posterior edge of the head. Morphologically, *S. changlensis* differs from its congeners by having pelvic‐fin rays that do not reach the anus when extended, 42–46 lateral line scales, and a posterior operculum margin reaching the base of the pectoral fin at vertical. Molecular phylogenetic analyses using mitochondrial markers (*cytb* and ND4), genetic distances, and geometric morphometric analysis further confirmed *S. changlensis* as a distinct species. The description of this new species contributes to the understanding of cave‐dwelling fish diversity in China and underscores the importance of further exploration of stygomorphic species across this poorly explored karstic landscape.

## Graphical abstract

The karst landscapes of southwestern China, one of the world's largest, support a high diversity of cavefish, including the *Sinocyclocheilus* genus, which has 84 species adapted to subterranean life. Here, we describe a new species, *Sinocyclocheilus changlensis*, distinguished by traits such as eyelessness, unpigmented scales, and unique morphological features. Molecular and morphological analyses confirm its distinctiveness, highlighting the necessity for further research on China's understudied cavefish diversity.

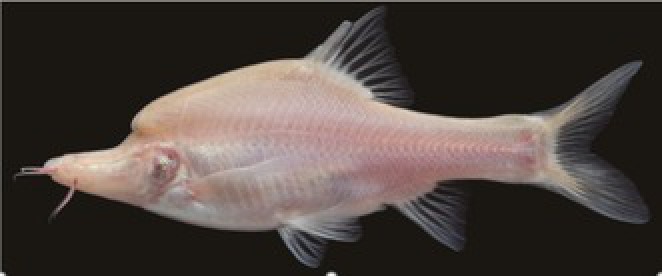

## 1 Introduction

The karts landscapes of China span over 1,900,000 km^2^, including 907,000 km^2^ of surface limestone. These regions represent one of the most significant karst distributions in the world and are among the most threatened biodiversity hotspots (Duan et al. 2021; Yuan 2002). The southwestern region, notably Yunnan, Guizhou, and Guangxi, spans over 620,000 km^2^ of karst terrain, making it an important region for studying cave‐adapted species, particularly cavefish (Huang et al. 2008; Zhao et al. 2011; Zhang and Zhu 2012). This region is home to the world's most diverse array of cavefish, including the *Sinocyclocheilus* genus, the most species‐rich cavefish taxon in the world (Lunghi et al. 2019; Xing et al. 2016; Mao et al. 2025).

*Sinocyclocheilus* has attracted scientific interest since 1904, when C. T. Regan first collected a specimen from Dianchi Lake in Yunnan, initially identified as *Barbus grahami*, later reclassified as *Sinocyclocheilus grahami* (Wu 1977). Further discoveries by Fang Bingwen in 1936, particularly *Sinocyclocheilus tingi* from Fuxian Lake, established the genus as a distinct group within Chinese cavefish (Fang 1936; Shan and Yue 1994; Zhao and Zhang 2009). Despite the early pioneering taxonomic work, due to the vastness of the karstic landscape, many new species are still being described (Mao et al. 2021). Hence, exploration for new species of cavefishes continues in the region.

*Sinocyclocheilus* is endemic to China and consists of 84 predominantly stygomorphic species (Chen et al. 2025; Fan et al. 2024; Luo et al. 2023; Mao et al. 2021; Shao et al. 2024; Wen et al. 2022; Xiao et al. 2025; Xu et al. 2023). Except for *Sinocyclocheilus sanxiaensis*, which inhabits the Three Gorges Reservoir, Hubei Province, all species are confined to the main karst regions of Guangxi, Guizhou, and Yunnan, residing in environments such as dragon pools, sinkholes, and underground rivers (Jiang et al. 2019; Zhao and Zhang 2009; Zhao et al. 2011). These environments have shaped the morphological diversity observed within the genus (Ma et al. 2019, 2020; Mao et al. 2021).

Despite considerable taxonomic attention, uncertainties remain regarding species delineation within *Sinocyclocheilus*, particularly in cases where morphological traits are subtle or inconsistent across populations (Mao et al. 2021; Zhao and Zhang 2009). Many species exhibit purportedly adaptive traits such as reduced or absent eyes, lack of pigmentation, and scaleless bodies (Zhao and Zhang 2009; Zhao et al. 2011). Some species have also evolved unique morphological features, such as horn‐like structures and humped backs (Zhao et al. 2011). However, the morphological diversity within the genus presents challenges for accurate species identification and classification (Wen et al. 2022; Xu et al. 2023).

To overcome these challenges, researchers working on *Sinocyclocheilus* within the past decade have used integrative taxonomic methods—morphological methods supported by molecular phylogenies (Jiang et al. 2019; Liu et al. 2018; Liu et al. 2025; Luo et al. 2023; Xu et al. 2023). This study also employs integrative taxonomic methods to resolve the systematic relationships and clarify species boundaries within *Sinocyclocheilus*.

During our explorations in the region, we collected specimens of a completely scaled, unpigmented *Sinocyclocheilus* population, exhibiting a bifurcated, horn‐like cranial protuberance that arises from the posterior head region and arches forward over the head, in central Guangxi, South China. Through molecular phylogenetic analyses and morphological comparisons, we confirm that these specimens represent a previously undescribed species of *Sinocyclocheilus*.

These results will contribute to a more refined understanding of *Sinocyclocheilus* taxonomy, clarifying species diversity within the genus. Accurate species identification is essential for conservation management and for maintaining the biological heritage of China's vulnerable karst ecosystems.

## 2 Materials and Methods

### 2.1 Specimen Sampling

**FIGURE 1:**
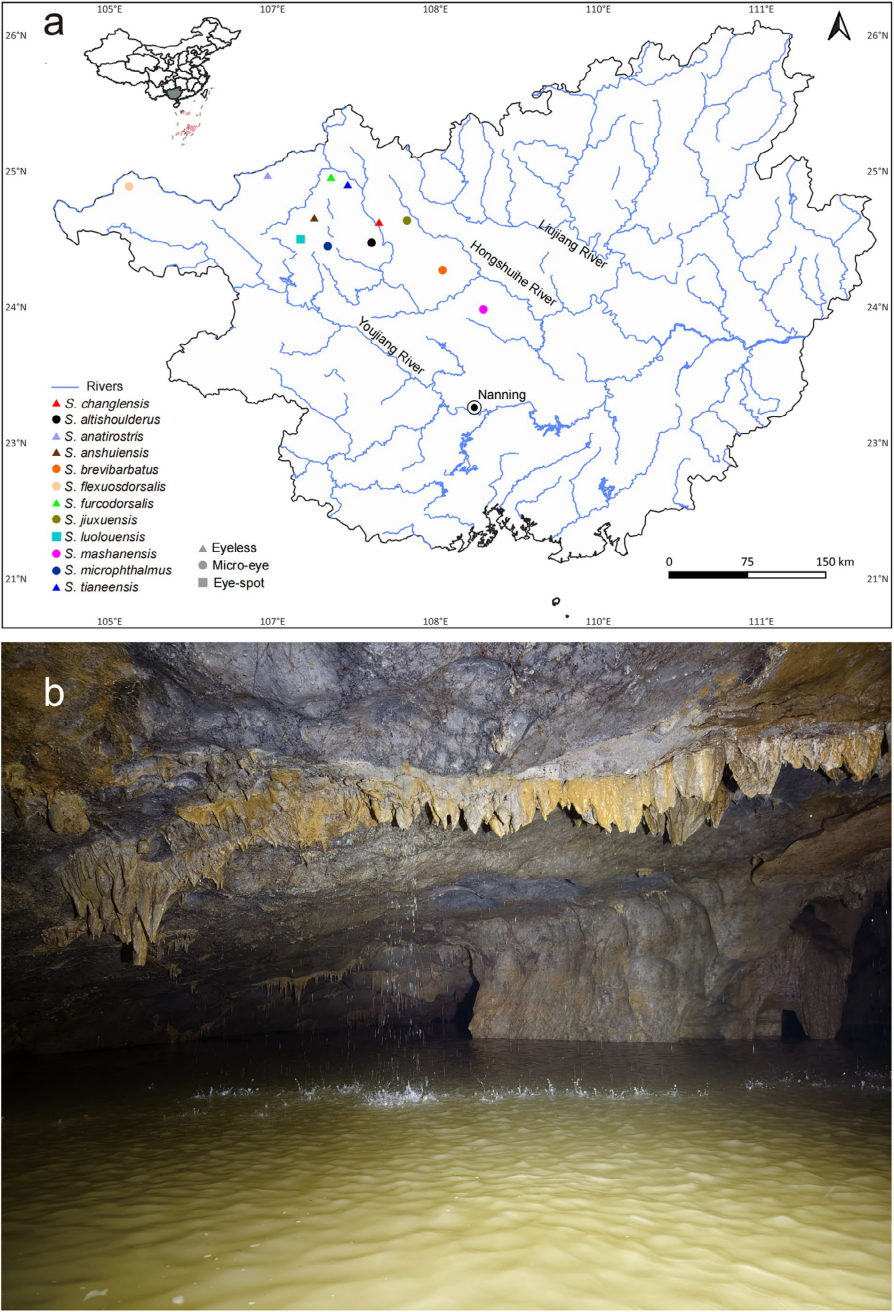
(a) Sampling collection localities of *Sinocyclocheilus changlensis*, new species, and its relatives in this study. Triangles represent species that are eyeless, circles represent species that are micro‐eyed, squares represent species that are eye‐spot. (b) Habitat of *Sinocyclocheilus changlensis*.

The treatment of experimental animals in this study fully complies with the Chinese Animal Welfare Law (GB/T 35892‐2018). Between 2020 and 2024, we sampled the *Sinocyclocheilus* species in Guangxi Zhuang Autonomous Region (Figure 1). Depending on the cave type, we used different collection methods, including direct hand nets, trap nets, and, in a few instances, cave‐diving. The following species co‐inhabited the Hongshui river system, where the new species was found: *S. furcodorsalis* (Chen et al. 1997), *S. tianeensis* (Li, Xiao, et al. 2003), *S. simengensis* (Wu et al. 2018), *S. altishoulderus* (Li and Lan 1992), *S. jiuxuensis* (Li, Lan, et al. 2003), *S. brevibarbatus* (Zhao et al. 2010), *S. mashanensis* (Wu et al. 2010), *S. flexuosdorsalis* (Zhu and Zhu 2012), *S. anatirostris* (Lin and Luo 1986), *S. anshuiensis* (Gan et al. 2013), *S. microphthalmus* (Li 1989), *S. luolouensis* (Lan et al. 2013), *S. macrophthalmus* (Zhang and Zhao 2001), *S. donglanensis* (Zhao et al. 2006), and *S. lingyunensis* (Li et al. 2000). From these, we retained the stygomorphic species and excluded the non‐stygomorphic species (*S. macrophthalmus, S. donglanensis*, and *S. lingyunensis* ) from our morphological comparisons.

We collected altogether 58 specimens of *Sinocyclocheilus*, among which 14 specimens represented an undescribed species from Changle Township in Donglan County; *S. furcodorsalis* (*n* = 7) from Bala Township in Tiane County; *S. tianeensis* (*n* = 7) from Bamu Township in Tiane County; *S. altishoulderus* (*n* = 3) from Sanshi Town in Donglan County; *S. mashanensis* (*n* = 4) from Guzhai Township in Mashan County; *S. jiuxuensis* (*n* = 2) from Jiuxu Township in Hechi City; *S. brevibarbatus* (*n* = 5) from Gaolin Township in Duan County; *S. flexuosdorsalis* (*n* = 3) from Tianshengqiao Township in Longlin County; *S. anatirostris* (*n* = 2) from Youping Township in Leye County; *S. microphthalmus* (*n* = 5) from Nashe Township in Bama County; *S. anshuiensis* (*n* = 5) from Jinya Township in Fengshan County; *S. luolouensis* (*n* = 1) from Luolou Township in Lingyun County. Due to the rarity and inaccessibility of the deep cave habitats, only a few specimens were collected. The live fish were first anesthetized with MS‐222, and then the right pelvic fins of some fish were dissected and placed in 95% ethanol. These specimens were then placed in a 10% formaldehyde solution for fixation. Finally, formalin‐fixed specimens were transferred to 75% ethanol for long‐term preservation. All specimens were deposited in Guangxi University (GXU), Nanning City, Guangxi Zhuang Autonomous Region.

### 2.2 Specimens Examined

*Sinocyclocheilus furcodorsalis* (*n* = 7): GXU2020000010‐14, GXU2020000058‐59, 7 specimens, 65.26–83.83 mm SL; Tiane County, Hechi City, Guangxi Zhuang Autonomous Region, China. These specimens are stored at Guangxi University, East Daxue Road, Xixiangtang District, Nanning, Guangxi, China. KIZ1993001542, 83.77 mm SL; Tiane County, Hechi City, Guangxi Zhuang Autonomous Region, China. These specimens are stored at the Kunming Institute of Zoology, CAS, Longxin Road, Panlong District, Kunming, Yunnan, China.

*Sinocyclocheilus tianeensis* (*n* = 7): GXU2020000015‐19, GXU2020000056‐57, 7 specimens, 83.76–112.57 mm SL; Tiane County, Hechi City, Guangxi Zhuang Autonomous Region, China. These specimens are stored at Guangxi University, East Daxue Road, Xixiangtang District, Nanning, Guangxi, China.

*Sinocyclocheilus brevibarbatus* (*n* = 5): GXU2020000028, GXU2020000031‐34, 5 specimens, 107.53–135.90 mm SL; Duan County, Hechi City, Guangxi Zhuang Autonomous Region, China. These specimens are stored at Guangxi University, East Daxue Road, Xixiangtang District, Nanning, Guangxi, China.

*Sinocyclocheilus mashanensis* (*n* = 4): GXU2020000001‐04, 4 specimens, 113.84–151.52 mm SL; Mashan County, Nanning City, Guangxi Zhuang Autonomous Region, China. These specimens are stored at Guangxi University, East Daxue Road, Xixiangtang District, Nanning, Guangxi, China.

*Sinocyclocheilus altishoulderus* (*n* = 3): GXU2020000020‐22, 3 specimens, 129.26–137.16 mm SL; Donglan County, Hechi City, Guangxi Zhuang Autonomous Region, China. These specimens are stored at Guangxi University, East Daxue Road, Xixiangtang District, Nanning, Guangxi, China.

*Sinocyclocheilus jiuxuensis* (*n* = 2): GXU2020000029‐30, 2 specimens, 133.68–134.80 mm SL; Jinchengjiang District, Hechi City, Guangxi Zhuang Autonomous Region, China. These specimens are stored at Guangxi University, East Daxue Road, Xixiangtang District, Nanning, Guangxi, China.

*Sinocyclocheilus flexuosdorsalis* (*n* = 3): GXU2020000056‐58, 3 specimens, 85.56‐92.70 mm SL; Longlin County, Baise City, Guangxi Zhuang Autonomous Region, China. These specimens are stored at Guangxi University, East Daxue Road, Xixiangtang District, Nanning, Guangxi, China.

*Sinocyclocheilus microphthalmus* (*n* = 5): GXU2020000063‐67, 5 specimens, 108.59‐113.72 mm SL; Bama County, Hechi City, Guangxi Zhuang Autonomous Region, China. These specimens are stored at Guangxi University, East Daxue Road, Xixiangtang District, Nanning, Guangxi, China.

*Sinocyclocheilus anshuiensis* (*n* = 5): GXU2020000068‐72, 5 specimens, 63.72–87.51 mm SL; Fengshan County, Hechi City, Guangxi Zhuang Autonomous Region, China. These specimens are stored at Guangxi University, East Daxue Road, Xixiangtang District, Nanning, Guangxi, China. KIZ20125501, 1 specimen, 82.20 mm SL; Lingyun County, Baise City, Guangxi Zhuang Autonomous Region, China. This specimen is stored at the Kunming Institute of Zoology, CAS, Longxin Road, Panlong District, Kunming, Yunnan, China.

*Sinocyclocheilus luolouensis* (*n* = 1): GXU2020000073, 1 specimen, 106.47 mm SL; Lingyun County, Baise City, Guangxi Zhuang Autonomous Region, China. These specimens are stored at Guangxi University, East Daxue Road, Xixiangtang District, Nanning, Guangxi, China.

*Sinocyclocheilus anatirostris* (*n* = 2): GXU2020000074‐75, 2 specimens, 66.16–73.11 mm SL; Leye County, Baise City, Guangxi Zhuang Autonomous Region, China. These specimens are stored at Guangxi University, East Daxue Road, Xixiangtang District, Nanning, Guangxi, China.

### 2.3 Morphological Analyses

Morphometric data were collected from 58 well‐preserved specimens of *Sinocyclocheilus* (Table A1). Point‐to‐point measurements were made on the left side of the specimen using an electronic vernier caliper with an accuracy of 0.1 mm. All morphometric measurements, counts, and terminology follow Shao et al. (2024) and Zhao et al. (2006). All morphometric measurements were converted to standard length (SL) percentages, rounded to 0.1%, and subjected to a logarithmic transformation for traditional morphometric (TM) analysis. Because some species are blind, the four linear features related to the eyes were excluded from the TM analysis. An exploratory principal component analysis (PCA) was conducted on the linear data to visualize general patterns of morphological variation among species and to identify morphometric variables that distinguish species in multivariate space. To test for significant differences in overall body size between species, a multivariate analysis of variance (MANOVA) was performed using the first three principal components (PCs) as dependent variables and species as the independent (predictor) variable. PCA and MANOVA analyses, including post hoc comparisons, were conducted using the software PAST (v. 4.04) (Hammer et al. 2001).

From a morphological perspective, *S. changlensis* and seven other species of *Sinocyclocheilus* (*S. furcodorsalis, S. tianeensis, S. altishoulderus, S. jiuxuensis, S. brevibarbatus, S. mashanensis*, and *S. flexuosdorsalis*) belong to the *S. angularis* group (Zhao and Zhang 2009). Previous phylogenetic studies have indicated that these seven *Sinocyclocheilus* species are closely related (Fan et al. 2024; Luo et al. 2023; Mao et al. 2022). Based on this, we selected a dataset of 45 specimens for the PCA and MANOVA analyses.

Nano‐computed tomography (nano‐CT) scanning and three‐dimensional (3D) reconstruction of specimens representing *S. furcodorsalis* , *S. tianeensis*, and *S. changlensis* were performed using the Tomography and Digital Imaging system (GE phoenix v|tome|x m 300 & 180CT) at the Key Laboratory of Vertebrate Origin and Human Evolution, Institute of Vertebrate Paleontology and Paleoanthropology (IVPP), Chinese Academy of Sciences. The entire CT scan of each specimen was conducted with an operating voltage of 80 kV and a current of 80 mA. Following a 360° rotational scan, the data were reconstructed into 1536 slices with a resolution of 4096 × 4096 pixels and an image resolution of 12.5 μm. Virtual model reconstruction was performed using Volume Graphics Studio 3.4.0.

In addition, X‐ray scanning reconstructions of vertebral counts were taken for *S. furcodorsalis* (*n* = 6), *S. tianeensis* (*n* = 6), and *S. changlensis* (*n* = 6) using a medical diagnostic X‐ray tube assembly system (TOSHIBA ROTANODE E7843X). The total number of vertebrae was determined by counting from the first free vertebra to the last half‐vertebra.

### 2.4 Geometric Morphometric Analysis

To assess variation in overall body shape along the lateral profile among these species, we constructed a database for landmark‐based geometric morphometrics using images of adult *Sinocyclocheilus* specimens. These images were sourced from our own photographs, taken with a scale for accuracy, as well as reference images from one of our previous studies—Mao et al. (2021). Measurements of the specimens were taken exclusively from the left side (lateral aspect) of the fish. The final dataset consisted of 71 images from 11 species. Geometric morphometric analysis was conducted using *tpsDig* v. 2.16 (Rohlf 2010a) with each image digitized based on 15 landmarks and 180 sliding semilandmarks (Mao et al. 2021). Each semilandmark was defined based on the tangent vector of its position profile, with semilandmarks grouped into curves to outline the body shape. The data were then converted into isometric landmarks and designated as semilandmarks using *tpsUtil* v. 1.46 (Rohlf 2010b). To correct for potential bending effects in the images, we applied the *unbend* function in *tpsUtil*.

The generalized Procrustes analysis, implemented in the software *tpsRelw* v.1.67 (Rohlf 2015), was used to align shape data, eliminating non‐shape variations caused by specimen size, position, and orientation. To examine population structure and differentiation in multivariate body shape data, canonical variate analysis (CVA) was performed on Procrustes shape coordinates in the lateral view. By identifying shape features that maximize differentiation between predefined groups (as opposed to variations within groups), CVA is commonly used to assess group separation, thereby aiding in species identification.

The software *MorphoJ v.1.08* was used to evaluate the statistical significance of differences in mean shape pairs, based on 10,000 Procrustes‐based permutations (Klingenberg 2011).

### 2.5 DNA Extraction, PCR, and Sequencing

DNA was extracted from 95% ethanol‐fixed fin tissue using the DNeasy Blood and Tissue Kit (Qiagen Inc., Valencia, CA) following the manufacturer's protocols. Fragments of the Cytochrome b (*cytb*) and NADH dehydrogenase subunit 4 (ND4) genes were amplified by polymerase chain reaction (PCR) using the primers DonThr R (5′‐ACC TCC GAT CTT CGG ATT ACA AGA CCG‐3′) and DonGlu F (5′‐AAC CAC CGT TGT ATT CAA CTA CAA‐3′) for *cytb* following Sudasinghe et al. (2018), ND4F (5′‐AAC AAG ACC TCT GAT TTC GGC TCA‐3′) and ND4R (5′‐TAG CTT CCA CTT GGA TTT GCA CC‐3′) for ND4 following Li et al. (2008).

Each PCR reaction, conducted in a 25‐μL volume, consisted of 12.5‐μL GoTaq Green Master Mix, 9.7‐μL Nuclease‐Free Water, 0.4‐μL of each primer, and 2‐μL DNA extract. The PCR conditions for the *cytb* followed an initial denaturation at 94°C for 2 min, followed by 35 cycles of denaturation at 94°C for 1 min, annealing at 48°C for 1 min, extension at 72°C for 1.5 min, and a final extension of 72°C for 5 min; for ND4, an initial denaturation at 95°C for 3 min, followed by 35 cycles of denaturation at 94°C for 0.5 min, annealing at 51°C for 0.5 min, extension at 72°C for 1.5 min, and a final extension of 72°C for 8 min (Li et al. 2008; Sudasinghe et al. 2020). PCR products were visualized by electrophoresis on a 1.0% agarose gel and then purified using a PCR purification kit (Qiagen) and sequenced in both directions with the corresponding primers by a commercial sequencing company. All newly‐generated sequences have been submitted to the GenBank (Table A2).

### 2.6 Phylogenetic Analyses

We used a total of 250 mitochondrial gene sequences for molecular analyses (130 *cytb* sequences and 120 ND4 sequences). The sample Liu044 was only sequenced for the *cytb* sequence, while the other 23 samples were sequenced for both these mitochondrial genes. Another 197 *Sinocyclocheilus* sequences were downloaded from GenBank, and we selected *Linichthys laticeps* and *Cyprinus carpio* as the outgroup (Table A2).

All sequences were revised manually and were aligned with MAFFT v.7.505 (Katoh and Standley 2013) using the “‐‐auto” strategy and normal alignment mode in PhyloSuite v.1.2.2 (Zhang et al. 2020). Alignment results were checked by eye and manually trimmed. The complete concatenated dataset included 130 samples of 65 *Sinocyclocheilus* species with a total alignment length of 2142 bp. Phylogenetic trees were constructed using maximum likelihood (ML) and Bayesian inference (BI) methods. The best‐fitting nucleotide substitution model for each dataset according to the Bayesian Information Criterion with Partition Model using *ModelFinder* v.1.6.8 (Kalyaanamoorthy et al. 2017) as implemented by *PhyloSuite* 1.2.2, the first, second, and third codons model of *cytb* and ND4 genes were defined within Partition Mode. The ML tree was conducted in IQ‐TREE v.1.6.8 (Nguyen et al. 2015) as implemented by *PhyloSuite* v.1.2.2 with 5000 ultrafast bootstrap replicates and with selected K2P+I+G4, TN+F+I+G4, TIM2+F+I+G4 model for the first, second, and third codons of the dataset. BI was performed in *MrBayes* v.3.2.6 (Ronquist et al. 2012) under the selected K2P+I+G4, GTR+F+I+G4, GTR+F+I+G4 model for three codons, using the MCMC method (four chains simultaneously run for 1 × 10^7^ generations) to calculate posterior probability, with tree sampling frequency set to 1 per 1000 cycles and the initial 25% of the sampled data discarded as burn‐in, resulting in a potential scale reduction factor of < 0.01. Nodes in the trees were considered well supported when Bayesian posterior probabilities were ≥ 0.95 and the ML ultrafast bootstrap value was ≥ 95%. Uncorrected *p*‐distances (1000 replicates) based on *cytb* and ND4 genes were calculated in MEGA 11.0 (Tamura et al. 2021).

## 3 Results

### 3.1 Morphological Analyses

**FIGURE 2:**
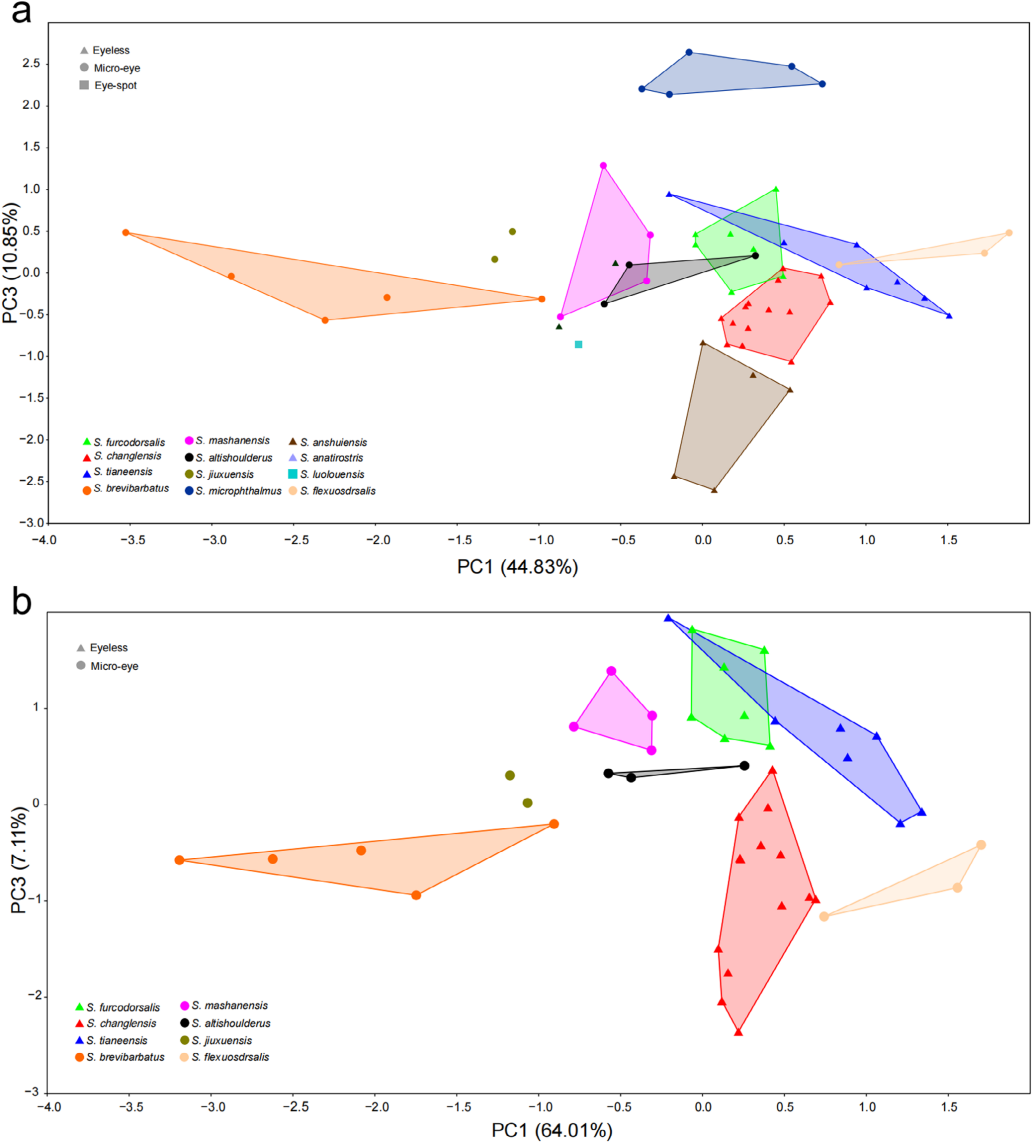
Results of principal component analysis (PCA) of overall body shape variation based on linear data. (a) Data set of 58 specimens from 12 species, including new species. (b) Data set of 45 specimens from eight species, including new species.

PCA of the dataset comprising 58 specimens from 12 species, based on 24 logarithmically transformed features, revealed that the first three PCs explained 74.19% of the total variance. Specifically, PC1 accounted for 44.83%, PC2 for 18.51%, and PC3 for 10.85% (Figure 2a). In the scatter plot of PC1 versus PC3, *S. changlensis* formed a distinct cluster with two homologous species ( *S. furcodorsalis* , and *S. anshuiensis*) along the PC3 axis, while it also clustered separately from the other eight homologous species along the PC1 axis (Figure 2a). Traits with high loadings on PC3 included dorsal‐fin length, pectoral‐fin length, and pelvic‐fin length, whereas traits with high loadings on PC1 included maxillary barbel length and rictal barbel length. A slight overlap was observed between *S. furcodorsalis* and *S. changlensis* along the PC3 axis (Figure 2a).

PCA of the dataset comprising 45 specimens from eight species, based on the same 24 logarithmically transformed features, showed that the first three components explained 80.64% of the total variance. PC1 accounted for 64.01%, PC2 for 9.52%, and PC3 for 7.11% (Figure 2b). PC1 was primarily associated with changes in maxillary barbel length and rictal barbel length, which exhibited the highest variation. PC3 was mainly related to pelvic‐fin base length and the width between the posterior nostrils. In the scatter plot, *S. changlensis* formed a distinct cluster from *S. furcodorsalis* along the PC3 axis and from *S. tianeensis* along the PC1 axis (Figure 2b). For both the 58‐specimen and 45‐specimen datasets, MANOVA results indicated statistically significant differences among species (Tables 1 and 2).

**TABLE 1:** Results of MANOVA on linear trait data of dataset of 58 specimens from 12 species.

	Test statistic	df1	df2	*F*	*p*
Wilks' lambda	2.26E‐09	250	230	6.875	1.45E‐43
Pillai trace	7.892	250	310	4.642	3.00E‐36

**TABLE 2:** Results of MANOVA on linear trait data of data set of 45 specimens from eight species.

	Test statistic	df1	df2	*F*	*p*
Wilks' lambda	4.29E‐07	150	72.4	5.418	1.54E‐13
Pillai trace	5.259	150	96	4.541	4.48E‐14

**FIGURE 3:**
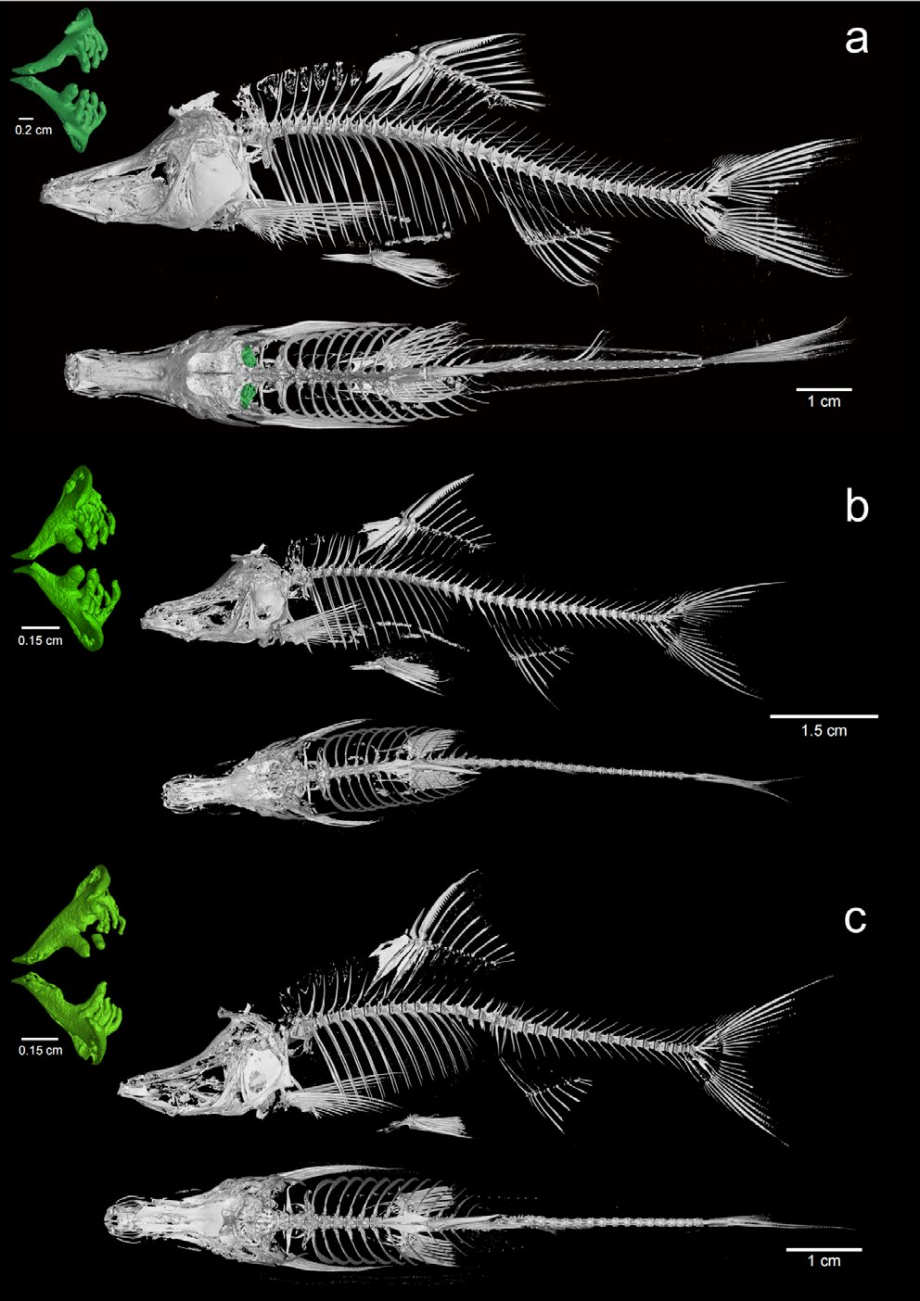
Micro‐CT graph and reconstructed pharyngeal dentition. (a) *Sinocyclocheilus changlensis*, new species; (b) *Sinocyclocheilus furcodorsalis* ; (c) *Sinocyclocheilus tianeensis*.

CT images revealed that the vertebral count of *S. changlensis* was 4 + 35, with a pharyngeal teeth pattern of 1,3,4–3,3,1. In comparison, *S. furcodorsalis* had a vertebral count of 4 + 34 and a pharyngeal teeth pattern of 2,3,4–4,3,2, while *S. tianeensis* had a vertebral count of 4 + 32 and the same pharyngeal teeth pattern (Figure 3). X‐ray scans further confirmed vertebral counts of 4 + 35 (*n* = 5) and 4 + 36 (*n* = 2) for *S. changlensis*, 4 + 32 (*n* = 1), 4 + 33 (*n* = 4), and 4 + 34 (*n* = 1) for *S. furcodorsalis* , and 4 + 32 (*n* = 2) and 4 + 34 (*n* = 4) for *S. tianeensis*. Dissections revealed that the first gill arch of *S. changlensis* had 11 outer rakers, whereas both *S. furcodorsalis* and *S. tianeensis* had 9 outer rakers.

### 3.2 Geometric Morphometric Analysis

**FIGURE 4:**
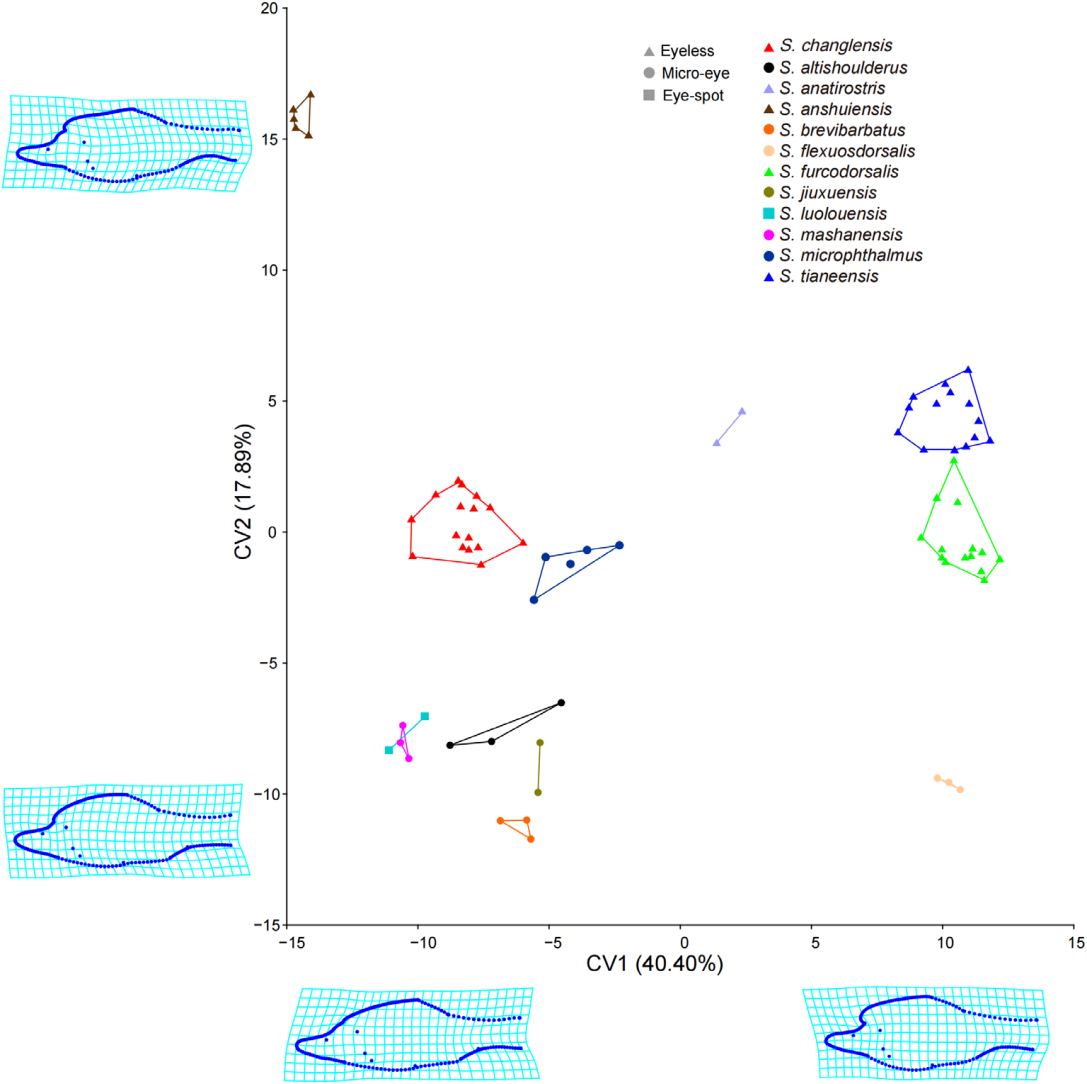
Results from canonical variate analysis (CVA) of overall body shape variation based on landmark data in lateral profiles.

The overall morphological variation can be visualized through the projection constructed by CVA (Figure 4). The lateral‐shape CVA indicates that 11 canonical variates (CVs) collectively explain 100% of the variation, with the first two accounting for 58.29% of the total shape variance.

The first CV, which explains approximately 44.40% of the variance, primarily differentiates species based on the height of the anterior dorsal and abdominal ridges. This variation is characterized by an increase in the prominence of the anterior dorsal and abdominal ridges from left to right, along with a more pronounced forward projection of the cephalic‐dorsal junction. These changes result in a shift in body shape from slender to broader, with forked angles becoming more distinct (Figure 4).

The second CV, accounting for about 17.89% of the variance, highlights interspecific differences in two key aspects: a progressive elongation and flattening of the head from bottom to top; a smoother dorsal profile, becoming increasingly streamlined from bottom to top (Figure 4).

All specimens of *S. changlensis* formed a distinct cluster that did not overlap with those of the other 11 *Sinocyclocheilus* species, indicating clear morphological differentiation (Figure 4).

### 3.3 Phylogenetic Analyses and Genetic Divergence

**FIGURE 5:**
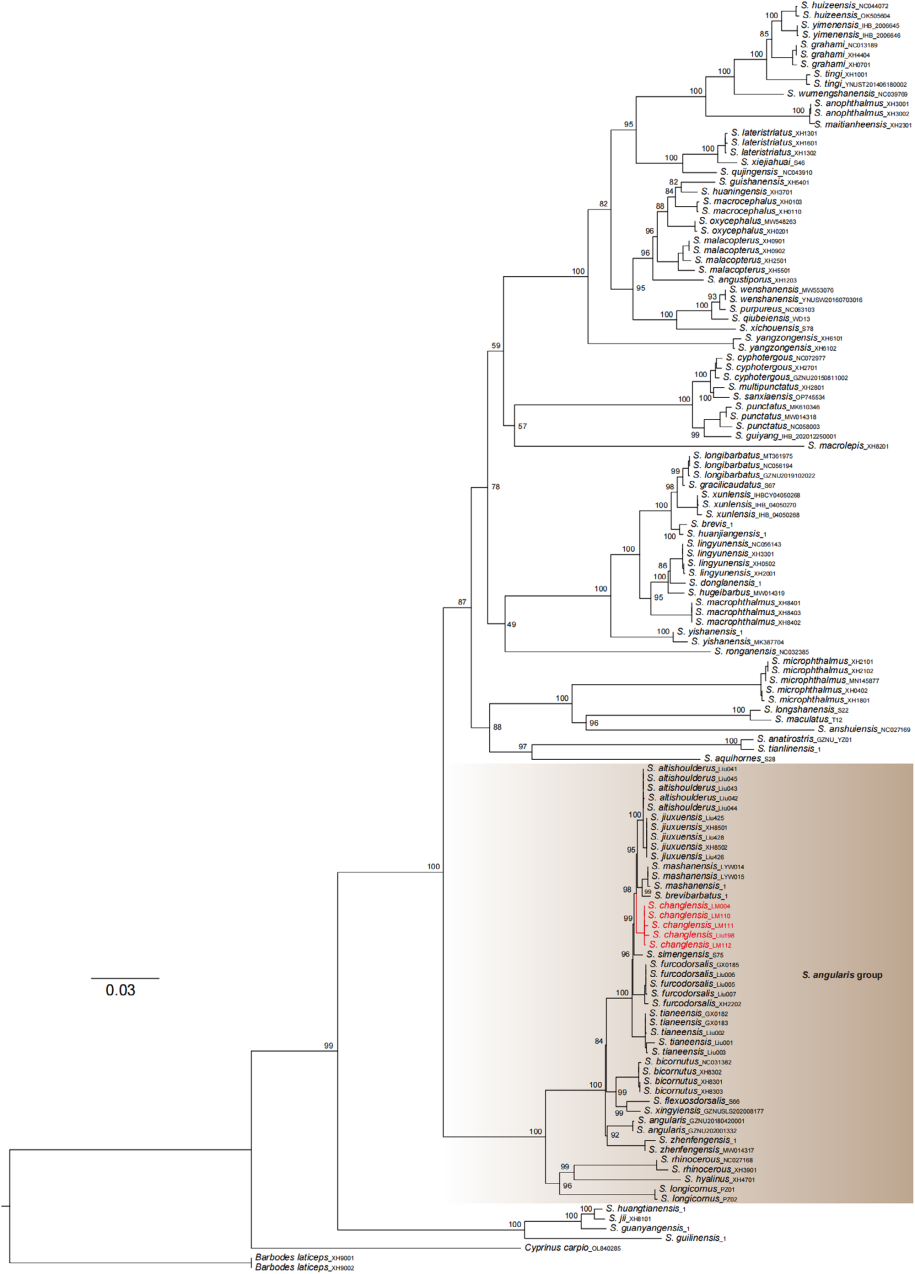
Molecular phylogenetic relationships of *Sinocyclocheilus*, based on maximum likelihood inference of the concatenated *cytb* + ND4 data set. The numbers near the nodes represent the ML bootstrap values, respectively.

**FIGURE A1:**
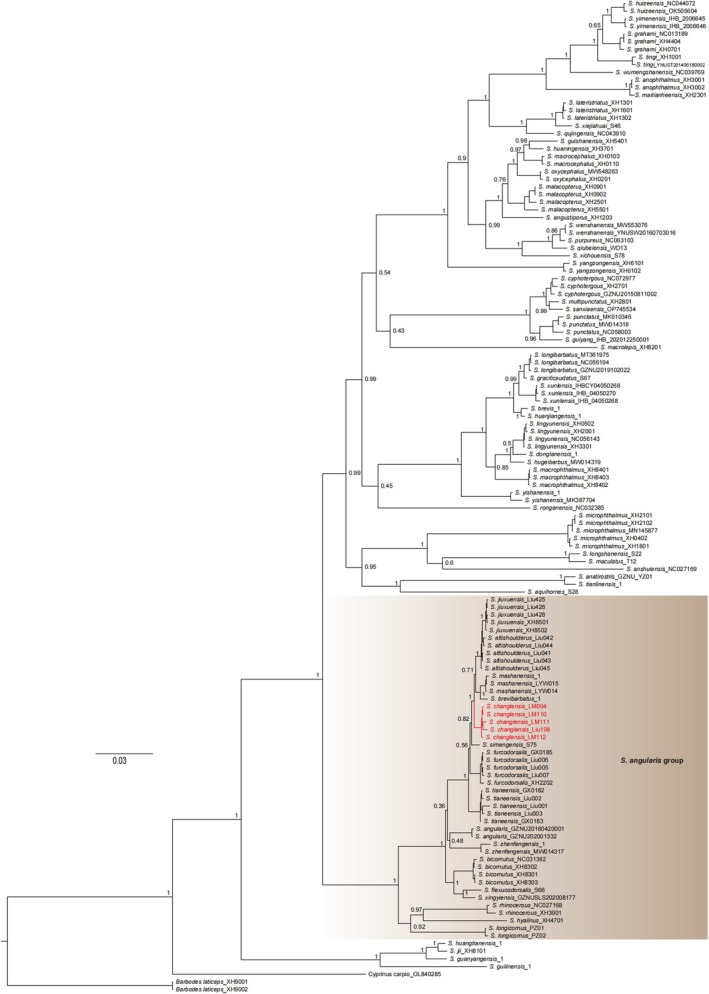
Molecular phylogenetic relationship of *Sinocyclocheilus*, based on Bayesian inference of the *cytb* + ND4 concatenated data set. The number of nodes near represents the Bayesian posterior probability.

ML and BI phylogenies were constructed based on two concatenated mitochondrial gene sequences, including 1110 bp *cytb* and 1032 bp ND4. The ML and the BI phylogenetic trees showed similar topology, only a slight difference in some branches (Figure 5; Figure A1). *Sinocyclocheilus changlensis* is resolved in the *S. angularis* group (Xu et al. 2023; Zhao and Zhang 2009) based on its phylogenetic position. The monophyly of the genus *Sinocyclocheilus* was strongly supported by both phylogenetic analyses. In ML and BI analyses, the *S. changlensis* formed a highly supported clade (98 in ML and 1 in BI) with *S. jiuxuensis*, *S. altishoulderus* , *S. brevibarbatus* , and *S. mashanensis*.

The *p*‐distances between *S. changlensis* and *S. jiuxuensis*, *S. altishoulderus* , *S. mashanensis*, *S. brevibarbatus* , *S. simengensis* all were less than 1.3% in *cytb*. The *p*‐distances between *S. changlensis* and the two species *S. furcodorsalis* and *S. tianeensis* were 1.7% and 2.4% in *cytb*, respectively. The *p*‐distances between *S. changlensis* and *S. altishoulderus* were 0.5%; *S. brevibarbatus* were 0.7%; *S. jiuxuensis* were 0.7%; and *S. mashanensis* were 0.9% in ND4. The *p*‐distances between *S. changlensis* and the two species *S. furcodorsalis* and *S. tianeensis* were 0.6% and 0.7% in ND4, respectively (Supporting Informations S1 and S2).

*Sinocyclocheilus changlensis* Liu, Mao & Yang, sp. nov.

**FIGURE 6:**
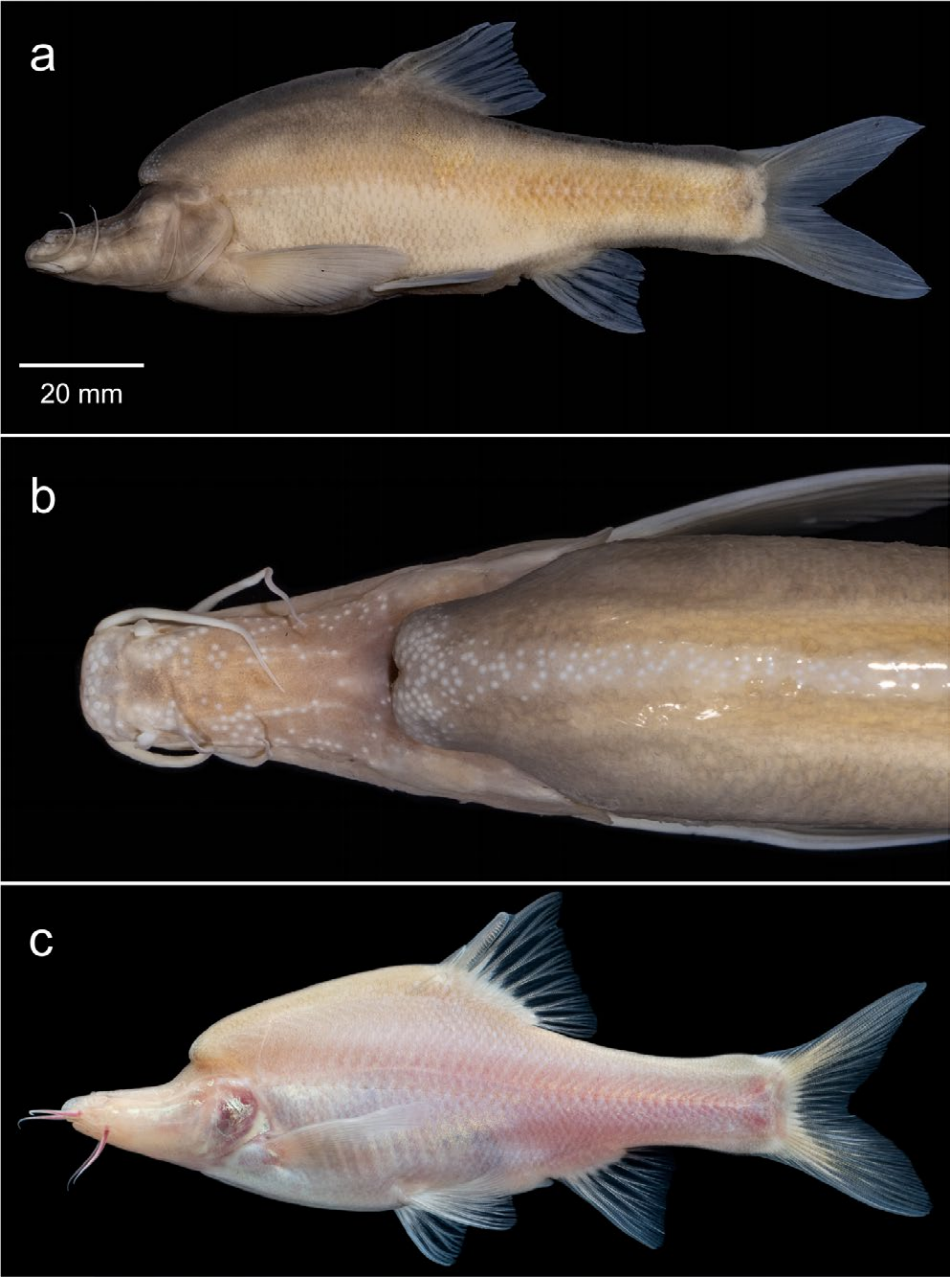
*Sinocyclocheilus changlensis*, GXU2020000041, HOLOTYPE, 137.8 mm SL. (a) Lateral view of preserved specimen; (b) dorsal view of head in preserved specimen; (c) live specimen.

Figure 6, Tables 3 and 4

**TABLE 3:** Comparison of morphological characteristics among the *Sinocyclocheilus changlensis* and its related species.

Character/species	*S. changlensis*	*S. furcodorsalis*	*S. tianeensis*	*S. jiuxuensis*	*S. brevibarbatus*	*S. mashanensis*	*S. altishoulderus*	*S. flexuosdorsalis*	*S. anshuiensis*	*S. microphthalmus*	*S. anatirostris*	*S. luolouensis*
Source	This study	This study	This study	This study	This study	This study	This study	This study	This study	This study	This study	This study
No. of specimens	14	7	7	2	5	4	3	3	5	5	2	1
Location	Hongshuihe	Hongshuihe	Hongshuihe	Hongshuihe	Hongshuihe	Hongshuihe	Hongshuihe	Hongshuihe	Hongshuihe	Hongshuihe	Hongshuihe	Hongshuihe
Dorsal‐fin rays	iii, 6–8	iii, 7	iii, 7–8	iii, 7	iii, 7	iii, 7–8	iii, 7	iii, 7–8	iii, 7	iii, 7–8	iii, 7–8	iii, 7
Anal‐fin rays	iii, 5	iii, 5	iii, 5	iii, 5	iii, 5	iii, 5	iii, 5	iii, 5	iii, 5	iii, 5	iii, 5	iii, 5
Pectoral‐fin rays	i, 13–15	i, 14–15	i, 14–16	i, 14	i, 13–14	i, 14	i, 14	i, 12–13	i, 11–12	i, 12–13	i, 10	i, 13
Pelvic‐fin rays	i, 7	i, 7–8	i, 7	i, 7	i, 7	i, 7–8	i, 7	i, 6–7	i, 7	i, 7	i, 6	i, 7
Lateral line scales	42–46	37–39	39–43	43–50	42–48	39–43	46–52	38–40	36–38	42–47	—	42
Scale rows above lateral line	11–14	11–13	11–13	12–14	11–13	11–13	12–13	11	7–8	9–12	—	9
Scale rows below lateral line	9–10	7–10	8–10	7–8	7–8	6–8	8–9	9–10	6	8–9	—	8
Body scales	Complete	Complete	Complete	Complete	Complete	Complete	Complete	Complete	Incomplete	Complete	Deficiency	Complete
Eyes	Eyeless	Eyeless	Eyeless	Micro	Micro	Micro	Micro	Micro	Eyeless	Micro	Eyeless	Eye‐spot

**TABLE 4:** Morphological comparison of *Sinocyclochelis changlensis*, *S. furcodorsalis* , *S. tianeensis*, *S. brevibarabtus*, *S. mashanensis*, *S. altishoulderus* , and *S. jiuxuensis*.

Measurements	*S. changlensis* (*N* = 14)		*S. furcodorsalis* (*N* = 7)		*S. tianeensis* (*N* = 7)		*S. brevibarbatus* (*N* = 5)		*S. mashanensis* (*N* = 4)		*S. altishoulderus* (*N* = 3)		*S. jiuxuensis* (*N* = 2)	
	Range	Mean ± SD	Range	Mean ± SD	Range	Mean ± SD	Range	Mean ± SD	Range	Mean ± SD	Range	Mean ± SD	Range	Mean ± SD
Standard length (mm)	73.9–137.8	104.4 ± 22.8	65.3–83.8	73.7 ± 6.5	83.8–112.6	97.1 ± 11.5	107.5–135.9	118.2 ± 12.1	113.8–151.5	133.2 ± 15.5	129.3–137.2	134.1 ± 4.3	133.7–134.8	134.2 ± 0.8
In % of standard length														
Body depth	27.8–33.5	30.7 ± 1.6	28.5–34.3	30.9 ± 2.1	30.5–36.0	32.8 ± 1.8	27.3–31.1	28.9 ± 1.7	28.5–32.2	30.2 ± 1.8	28.8–31.0	29.8 ± 1.1	28.0–30.4	29.2 ± 1.7
Predorsal length	56.6–60.6	58.7 ± 1.1	53.6–56.5	54.7 ± 1.1	56.6–58.4	57.4 ± 0.7	57.1–57.9	57.5 ± 0.3	56.3–59.7	57.7 ± 1.5	56.0–57.6	56.7 ± 0.8	56.1–57.7	56.9 ± 1.1
Dorsal‐fin base length	12.8–16.6	15.0 ± 1.3	14.8–17.6	15.9 ± 1.0	14.8–17.0	15.9 ± 0.7	11.8–13.7	12.9 ± 0.8	13.9–18.1	15.7 ± 1.8	15.2–15.6	15.3 ± 0.2	13.9–15.0	14.5 ± 0.8
Dorsal‐fin length	18.4–22.1	20.1 ± 1.1	18.7–22.4	20.3 ± 1.2	19.1–21.8	20.2 ± 1.2	19.8–22.2	20.8 ± 1.0	19.9–24.7	21.3 ± 2.2	18.6–19.5	19.1 ± 0.5	19.9–20.1	20.0 ± 0.2
Preanal length	71.5–77.2	73.8 ± 1.7	68.3–71.2	69.5 ± 1.1	68.9–72.3	70.4 ± 1.3	68.9–72.4	70.9 ± 1.4	69.3–70.8	70.2 ± 0.7	70.5–71.5	71.1 ± 0.5	69.1–68.2	68.1 ± 0.1
Anal‐fin base length	8.4–11.2	9.6 ± 0.8	9.8–11.5	10.7 ± 0.5	9.4–10.6	10.0 ± 0.4	8.0–8.8	8.3 ± 0.3	9.0–10.3	9.5 ± 0.6	9.1–9.8	9.4 ± 0.4	8.9–9.1	9.0 ± 0.1
Anal‐fin length	16.5–19.6	17.8 ± 0.9	15.3–19.4	17.2 ± 1.2	15.3–19.3	17.2 ± 1.4	15.8–19.8	17.4 ± 1.5	16.9–19.9	18.5 ± 1.2	13.9–15.1	14.6 ± 0.6	15.9–17.5	16.7 ± 1.1
Prepectoral length	28.7–33.4	30.9 ± 1.5	27.2–28.1	27.6 ± 0.4	27.9–29.9	28.9 ± 0.7	28.4–32.7	30.7 ± 1.6	29.2–31.1	30.2 ± 0.8	25.8–28.5	27.5 ± 1.5	28.5–30.0	29.3 ± 1.0
Pectoral‐fin base length	3.5–4.8	4.2 ± 0.4	3.7–5.0	4.4 ± 0.5	4.5–5.5	4.8 ± 0.3	4.1–4.6	4.3 ± 0.2	4.2–4.9	4.5 ± 0.3	4.1–4.8	4.5 ± 0.3	3.9–4.0	4.0 ± 0.1
Pectoral‐fin length	23.3–26.8	25.4 ± 1.0	20.5–23.8	22.1 ± 1.2	23.4–26.0	24.8 ± 1.0	22.2–24.7	23.3 ± 0.9	21.7–27.4	24.6 ± 2.8	21.1–23.5	22.5 ± 1.3	21.7–26.5	24.1 ± 3.4
Prepelvic length	50.3–54.0	51.6 ± 1.2	46.3–49.1	47.8 ± 1.0	48.2–51.5	50.3 ± 1.2	47.6–49.9	48.6 ± 0.9	46.0–48.1	47.3 ± 0.9	44.9–46.0	45.4 ± 0.5	47.0–47.6	47.3 ± 0.4
Pelvic‐fin base length	3.9–5.7	5.0 ± 0.5	5.2–6.0	5.6 ± 0.3	5.4–6.4	5.9 ± 0.3	3.6–4.7	4.0 ± 0.4	5.5–6.2	5.9 ± 0.3	5.0–5.2	5.1 ± 0.1	5.0–5.1	5.1 ± 0.1
Pelvic‐fin length	13.8–18.5	16.3 ± 1.3	14.8–17.9	15.8 ± 1.1	14.8–16.9	16.2 ± 0.8	17.0–20.5	18.4 ± 1.3	17.4–20.6	18.5 ± 1.5	15.2–17.2	16.5 ± 1.1	17.3–17.9	17.6 ± 0.4
Caudal peduncle length	19.2–21.5	20.1 ± 0.7	19.6–22.6	21.5 ± 1.2	21.2–22.2	21.8 ± 0.3	21.9–24.9	23.3 ± 1.2	21.2–24.6	22.7 ± 1.4	22.0–23.3	22.8 ± 0.7	24.5–24.7	24.6 ± 0.2
Caudal peduncle depth	11.8–14.0	12.8 ± 0.6	12.6–14.5	13.3 ± 0.7	12.8–14.7	13.7 ± 0.7	10.4–12.5	11.6 ± 0.8	12.4–13.8	13.4 ± 0.6	13.5–14.1	13.7 ± 0.3	11.6–11.8	11.7 ± 0.1
Head length	30.5–33.2	31.8 ± 0.9	27.9–30.1	29.1 ± 0.8	28.5–30.8	29.9 ± 0.8	29.7–33.1	30.9 ± 1.3	31.4–32.6	32.2 ± 0.5	27.4–28.7	28.2 ± 0.7	30.0–30.2	30.1 ± 0.1
Head depth	13.9–16.8	15.4 ± 1.0	12.4–15.1	14.2 ± 1.0	11.9–14.1	13.0 ± 0.8	15.5–17.3	16.4 ± 0.7	15.8–17.6	16.3 ± 0.9	14.4–15.8	15.1 ± 0.7	14.9–15.0	14.9 ± 0.1
Head width	13.3–16.6	14.7 ± 0.9	13.8–15.9	14.7 ± 0.7	14.4–16.4	15.6 ± 0.8	14.0–16.0	15.2 ± 0.8	15.8–16.6	16.4 ± 0.4	14.2–14.9	14.4 ± 0.4	13.0–14.2	13.6 ± 0.9
Snout length	/	/	/	/	/	/	9.2–11.8	10.0 ± 1.1	10.7–12.0	11.2 ± 0.6	9.5–10.8	10.2 ± 0.7	10.1–10.6	10.3 ± 0.3
Eyeball diameter	/	/	/	/	/	/	4.3–5.6	4.9 ± 0.6	3.4–4.2	3.7 ± 0.4	3.8–4.2	4.0 ± 0.2	2.7–3.2	2.9 ± 0.4
Eye diameter	/	/	/	/	/	/	8.5–9.5	9.2 ± 0.4	5.6–7.1	6.2 ± 0.6	7.0–7.6	7.4 ± 0.3	6.9–7.7	7.3 ± 0.6
Interorbital width	/	/	/	/	/	/	8.1–10.3	9.1 ± 0.9	6.8–9.0	8.2 ± 1.0	7.8–8.3	8.1 ± 0.2	8.0–8.0	8.0 ± 0.0
Prenostril length	4.8–5.8	5.5 ± 0.3	4.5–5.8	5.5 ± 0.4	4.8–6.5	5.5 ± 0.7	5.4–6.0	5.7 ± 0.3	5.3–7.5	5.9 ± 1.1	5.0–5.8	5.5 ± 0.4	5.2–6.0	5.6 ± 0.5
Width between posterior nostrils	4.9–6.2	5.8 ± 0.3	6.4–7.3	6.9 ± 0.4	5.2–7.0	6.2 ± 0.6	6.0–6.7	6.4 ± 0.3	6.5–7.6	7.1 ± 0.4	5.1–5.9	5.6 ± 0.4	6.2–6.5	6.3 ± 0.2
Upper jaw length	7.5–8.8	8.2 ± 0.4	7.4–7.9	7.6 ± 0.2	8.1–9.4	8.9 ± 0.5	7.9–9.7	9.0 ± 0.8	9.1–10.0	9.6 ± 0.4	7.6–7.8	7.6 ± 0.1	7.6–8.7	8.2 ± 0.8
Lower jaw length	6.1–8.0	6.9 ± 0.5	5.8–6.8	6.4 ± 0.3	6.5–7.9	7.1 ± 0.5	7.3–9.5	8.5 ± 0.9	7.3–8.8	8.3 ± 0.7	5.2–6.3	5.9 ± 0.6	6.9–7.4	7.2 ± 0.3
Mouth width	5.9–7.3	6.6 ± 0.4	5.8–6.6	6.2 ± 0.3	5.8–7.0	6.4 ± 0.5	7.7–9.0	8.3 ± 0.5	6.6–9.3	8.2 ± 1.3	5.9–6.9	6.6 ± 0.5	6.2–6.6	6.4 ± 0.3
Maxilla barbel length	10.2–12.3	11.3 ± 0.7	10.0–10.7	10.3 ± 0.3	9.0–15.8	13.2 ± 2.2	3.4–6.4	4.8 ± 1.3	6.4–8.6	7.6 ± 0.9	7.8–10.3	8.9 ± 1.3	6.3–6.7	6.5 ± 0.3
Rictal barbel length	9.0–11.9	10.1 ± 0.9	7.4–10.3	9.0–1.1	7.5–14.2	11.7 ± 2.5	2.8–7.9	5.2 ± 1.9	7.9–9.6	8.8 ± 0.7	7.0–9.7	8.0 ± 1.4	6.1–6.2	6.1 ± 0.1

Measurements	*S. flexuosdorsalis* (*N* = 3)		*S. anshuiensis* (*N* = 5)		*S. microphthalmus* (*N* = 5)		*S. anatirostris* (*N* = 2)		*S. luolouensis* (*N* = 1)
	Range	Mean ± SD	Range	Mean ± SD	Range	Mean ± SD	Range	Mean ± SD	Range
Standard length (mm)	85.6–92.7	88.1 ± 4.0	71.8–87.5	75.1 ± 8.6	108.6–113.7	111.4 ± 2.3	66.2–73.1	69.6 ± 4.9	106.5
In % of standard length									
Body depth	26.4–29.0	28.1 ± 1.4	25.3–29.3	27.3 ± 1.5	24.8–29.2	27.0 ± 1.8	20.6–24.3	22.4 ± 2.6	21.7
Predorsal length	54.6–57.2	55.7 ± 1.4	53.3–58.2	56.8 ± 2.1	49.6–52.0	50.4 ± 0.9	55.5–55.6	55.5 ± 0.1	55.8
Dorsal‐fin base length	17.2–18.9	18.0 ± 0.9	12.7–14.2	13.2 ± 0.6	15.7–17.0	16.3 ± 0.4	13.9–15.1	14.5 ± 0.8	12.0
Dorsal‐fin length	21.9–22.1	22.0 ± 0.1	18.4–22.6	20.1 ± 1.7	25.5–27.8	26.5 ± 0.8	23.1–26.2	24.6 ± 2.2	19.9
Preanal length	70.7–71.9	71.2 ± 0.6	71.8–76.1	74.6 ± 1.9	68.0–71.8	69.3 ± 1.3	73.4–76.9	75.1 ± 2.5	71.9
Anal‐fin base length	10.5–11.8	11.0 ± 0.7	8.5–9.8	9.1 ± 0.6	8.6–11.4	10.1 ± 1.0	9.4–11.0	10.2 ± 1.1	9.0
Anal‐fin length	18.1–21.3	19.3 ± 1.8	14.8–18.3	16.6 ± 1.5	19.1–22.2	20.6 ± 1.0	18.1–20.3	19.2 ± 1.5	17.8
Prepectoral length	28.4–32.4	30.4 ± 2.0	31.1–31.6	31.3 ± 0.2	25.6–27.1	26.3 ± 0.5	35.3–35.4	35.4 ± 0.1	30.7
Pectoral‐fin base length	4.3–5.1	4.6 ± 0.4	3.0–4.0	3.5 ± 0.5	3.6–4.2	4.0 ± 0.2	3.5–3.9	3.7 ± 0.3	4.1
Pectoral‐fin length	24.7–29.2	27.4 ± 2.4	17.8–23.0	20.5 ± 2.3	27.5–29.8	29.2 ± 0.8	25.5–25.8	25.7 ± 0.2	21.3
Prepelvic length	49.3–51.3	50.1 ± 1.1	50.9–54.6	52.5 ± 1.6	45.0–46.9	45.7 ± 0.7	54.5–55.2	54.9 ± 0.5	50.1
Pelvic‐fin base length	5.1–6.4	5.8 ± 0.6	3.1–4.7	4.1 ± 0.6	4.1–5.8	4.7 ± 0.6	3.9–4.3	4.1 ± 0.3	3.6
Pelvic‐fin length	17.9–20.3	19.0 ± 1.2	13.6–16.4	15.0 ± 1.3	21.8–23.2	22.3 ± 0.5	19.3–22.2	20.7 ± 2.1	17.9
Caudal peduncle length	19.7–20.4	20.1 ± 0.4	18.4–20.1	19.2 ± 0.7	21.7–23.6	22.5 ± 0.7	19.6–21.4	20.5 ± 1.3	20.8
Caudal peduncle depth	12.7–13.9	13.2 ± 0.6	10.9–12.7	11.5 ± 0.7	9.6–11.2	10.4 ± 0.6	7.9–9.4	8.6 ± 1.0	9.6
Head length	29.5–31.2	30.6 ± 1.0	30.4–32.7	31.5 ± 1.0	24.9–26.1	25.3 ± 0.4	32.8–34.7	33.8 ± 1.3	29.1
Head depth	15.4–16.4	15.8 ± 0.5	18.2–20.1	19.4 ± 0.8	13.2–14.8	14.0 ± 0.6	19.1–20.6	19.9 ± 1.1	15.9
Head width	13.5–13.8	13.6 ± 0.1	11.8–13.3	12.7 ± 0.6	12.2–13.3	12.7 ± 0.4	13.4–15.1	14.3 ± 1.2	13.3
Snout length	9.7–10.7	10.3 ± 0.5	/	/	7.6–8.5	8.0 ± 0.3	/	/	
Eyeball diameter	2.4–3.2	2.7 ± 0.4	/	/	1.3–1.4	1.3 ± 0.1	/	/	
Eye diameter	5.4–6.8	6.1 ± 0.7	/	/	4.3–4.5	4.4 ± 0.1	/	/	
Interorbital width	6.0–6.3	6.2 ± 0.2	/	/	6.7–7.5	7.1 ± 0.3	/	/	
Prenostril length	4.9–5.9	5.4 ± 0.5	3.9–4.8	4.4 ± 0.3	4.2–4.6	4.4 ± 0.1	5.3–5.3	5.3 ± 0.0	5.1
Width between posterior nostrils	4.9–5.4	5.2 ± 0.3	5.1–6.1	5.6 ± 0.4	5.4–6.9	6.2 ± 0.6	6.6–7.1	6.8 ± 0.3	5.6
Upper jaw length	7.5–8.8	8.2 ± 0.7	6.5–7.9	7.1 ± 0.5	5.4–6.5	6.1 ± 0.4	8.6–9.7	9.1 ± 0.8	7.2
Lower jaw length	6.7–8.0	7.1 ± 0.7	5.4–7.4	6.4 ± 0.7	5.1–5.6	5.3 ± 0.2	7.4–8.2	7.8 ± 0.6	6.9
Mouth width	5.8–6.8	6.3 ± 0.5	4.7–5.5	4.9 ± 0.3	4.9–5.5	5.2 ± 0.2	6.2–7.1	6.7 ± 0.6	5.8
Maxilla barbel length	11.2–16.7	14.5 ± 2.9	9.8–11.9	10.7 ± 0.9	8.0–11.7	9.8 ± 1.6	8.9–9.9	9.4 ± 0.7	7.1
Rictal barbel length	12.8–17.4	15.7 ± 2.6	8.7–11.2	9.8 ± 1.1	7.9–11.2	9.1 ± 1.3	7.1–7.6	7.4 ± 0.3	9.0

**Holotype**. GXU2020000041, 137.8 mm standard length (SL), adult male collected by Yewei Liu, Shipeng Zhou, Jiajun Zhou, and Chenghai Fu on August 17, 2020, from an underground river in a cave of Hongshuihe River of Pearl River Basin, Changle Township, Donglan County, Guangxi, China (24.5255° N, 107.4787° E, altitude 260 m above sea level).

**Paratypes**. GXU2020000035, GXU2020000037, GXU2020000040, GXU2020000046–GXU2020000055 73.9–127.4 mm SL (13 specimens), same data as the holotype.

**Diagnosis**

*Sinocyclocheilus changlensis* can be distinguished from all other congeners by the following combination of characters: eye absent (eyeless); pelvic‐fin rays tip not reaching the anus when pelvic‐fin rays are extended backward; having a forked horn‐like structure at the dorsal posterior edge of the head; albinotic body without pigmentation; lateral line scales 42–46; posterior margin of operculum close to the base of pectoral‐fin origin; a distinct hump in the predorsal profile; rostral barbel not reaching the depression after eye degeneration; maxillary barbel not reaching the posterior margin of preoperculum; pharyngeal teeth pattern 1,3,4–3,3,1; vertebrae 4 + 35–36; 11 outer rakers on the first gill arch.

**Description**

General body features as in Figure 6. Meristics and proportional measurements are provided in Tables 3 and 4, respectively. *Sinocyclocheilus changlensis* shares some morphological similarities with *S. furcodorsalis* , *S. tianeensis*, *S. altishoulderus* , *S. mashanensis*, *S. jiuxuensis*, and *S. brevibarbatus* . Body is laterally compressed. Greatest body depth is immediately anterior to the origin of the dorsal fin. Dorsal profile of the head is straight anteriorly and concave posteriorly. Predorsal profile of the body is convex, with a distinct hump along the back of the head, then sloping toward the dorsal‐fin origin. The frontal bone bulges after the bifurcation on the anterior part of the horn. Postdorsal profile of the body is concave. Ventral profile of the head is straight. Ventral profile of the body is slightly convex between the pectoral and pelvic‐fin origins, straight between the pelvic and anal‐fin origins, and slightly concave thereafter.

Head slightly compressed, laterally elongated, blunt in dorsal view. Mouth subterminal. Two pairs of barbels. Rostral barbel shorter than maxillary, extending beyond the origin of the maxillary barbel when adpressed, ending anterior to the depression after eye degeneration. Tip of the adpressed maxillary barbel not reaching the posterior edge of the preoperculum. Eyes absent, but small depressions on either side of the head or only on one side reflect the position of the degenerated eye.

Dorsal fin with three unbranched and 6 (7), 7 (6), 8 (1) branched rays. Last unbranched ray stiff, its posterior margin strongly serrated. Dorsal fin with its origin slightly posterior to pelvic‐fin origin, its distal margin straight. Anal fin with three unbranched and five branched rays, its distal margin straight. Origin of anal fin slightly closer to pelvic‐fin origin than to caudal‐fin base. Pelvic fin with single unbranched and seven branched rays. Origin of pelvic fin slightly closer to pectoral‐fin origin than anal‐fin origin. Adpressed pelvic fin not reaching vertical through anus. Pectoral fin with single unbranched and 13 (3), 14 (8), 15 (3) branched rays. Tip of adpressed pectoral fin reaching vertical through pelvic‐fin origin. Caudal fin with 8 + 7 (2), 8 + 8 (10), or 9 + 8 (2) branched rays, forked, lobes subequal, rounded distally. Eleven outer rakers (1) on first gill arch. Vertebrae 4 + 35 (4), 4 + 36 (2).

Lateral line complete, almost straight from operculum to caudal‐fin base, with 42 (5), 43 (3), 44 (4), 45 (1), 46 (1) scales on body. Median predorsal scales 26 (1), 27 (2), 28 (1), 32 (4), 33 (2), 34 (2), 36 (1), 39 (1). Lateral scale rows between lateral line scale row and middorsal scale row 11 (1), 12 (5), 13 (7), 14 (1). Lateral scale rows between lateral line scale row and midpelvic scale row 9 (4), 10 (10). Circumpeduncular scales 19 (1), 20 (1), 21 (3), 24 (1), 25 (2), 26 (2), 27 (2), 28 (1), 29 (1) (Table 3).

**Coloration**

For condition in life, see Figure 6c; in preservative see Figure 6a. In live specimens, the head and dorsal profile of the body are light brown, laterally silver. Barbels are red. In preservative, the head and body are grayish white. Barbels are white.

**Distribution and habitat**

This species has so far been recorded only from its type locality, a half‐open cave about 4 km from Changle Township, Donglan County, Guangxi, China. This cave is very close to the Hongshui River. There was faint light inside the cave. *Sinocyclocheilus changlensis* was only caught in a large pool nearest to the outside. Within this cave, *S. changlensis* co‐occurred with *Pterocryptis anomala* (Herre 1933), *Hongshuia megalophthalmus* (Chen et al. 2006), and *Schistura fasciolata*

(Nichols and Pope 1927).

**Etymology**

The specific epithet, changlensis, is derived from the name of the collection locality, Changle Township, Donglan County. The common name proposed for the new species is “长乐金线鲃” (Changle Golden‐line Barb).

## 4 Discussion

Morphological comparison and phylogenetic analysis support the distinct species status of *S. changlensis*. This species exhibits these key morphological traits that differentiate it from all other *Sinocyclocheilus* species, with the exception of those in the same *S. angularis* group. These traits include the presence of a short maxillary barbel not extending to the posterior edge of the preoperculum; a bulging frontal bone bifurcating anteriorly at the base of the horn; complete absence of eyes (blind); a distinct head shape protruding forward, resembling a duck's beak; and pectoral fins that, when adpressed, reach the vertical plane through the pelvic‐fin origin.

*Sinocyclocheilus microphthalmus* , *S. luolouensis*, *S. anshuiensis*, and *S. anatirostris* also belong to the Hongshui River system. *Sinocyclocheilus changlensis* can be distinguished from *S. microphthalmus* by the absence of eyes (vs. micro‐eyes), a forked horn‐like structure at the dorsal posterior edge of the head (vs. absent). It differs from *S. anshuiensis* by the presence of a forked horn‐like structure (vs. a smooth sarcoma forming at the junction of the head and back), rostral barbel shorter than maxillary barbel (vs. maxillary barbel shorter than rostral barbel). Additionally, *S. changlensis* is distinct from *S. luolouensis* by its forked horn‐like structure (vs. absent), absence of eyes (vs. presence of eye‐spot). Furthermore, it differs from *S. anatirostris* by the presence of a forked horn‐like structure (vs. a pair of small tubercles forming behind the frontal bone), rostral barbel shorter than maxillary barbel (vs. rostral barbel approximately as long as the maxillary barbel).

Among the 16 species within the same *S. angularis* group, *S. changlensis* can be distinguished from *S. longicornus* (Xu et al. 2023), *S. broadihornes* (Li and Mao 2007), *S. tileihornes* (Mao et al. 2003), *S. rhinocerous* (Li and Tao 1994), and *S. angularis* (Zheng and Wang 1990) by the absence of eyes (vs. presence of micro‐eyes), a forked horn‐like structure at the dorsal posterior edge of the head (vs. a single horn), and the presence of normal body scales (vs. scaleless or thinly scaled bodies). Furthermore, it differs from *S. flexuosdorsalis*, *S. xingyiensis* (Luo et al. 2023), *S. zhenfengensis* (Liu et al. 2018), and *S. bicornutus* (Wang and Liao 1997) by the absence of eyes (vs. micro‐eyes) and from *S. hyalinus* (Chen et al. 1994) by possessing a forked horn‐like structure (vs. single horn).

*Sinocyclocheilus changlensis* can also be morphologically distinguished from its closest relatives, including *S. brevibarbatus* , *S. jiuxuensis*, *S. altishoulderus* , *S. mashanensis*, and *S. simengensis*. It differs from *S. brevibarbatus* by the absence of eyes (vs. presence of micro‐eyes), the presence of a forked horn‐like structure (vs. absent), and a longer maxillary (10.2%–12.3% SL vs. 3.4%–6.4%) and rostral barbels (9.0%–11.9% SL vs. 2.8%–7.9%). Comparatively, *S. changlensis* can be distinguished from *S. simengensis* by the absence of eyes (vs. presence of micro‐eyes), lesser lateral line scales count (42–46 vs. 56–57), and a greater head length (30.5%–33.2% SL vs. 26.0%–26.3% SL). Additionally, *S. changlensis* is distinct from *S. altishoulderus* , *S. jiuxuensis*, and *S. mashanensis* by its forked horn‐like structure (vs. absent), absent eyes (vs. presence of micro‐eyes), and, in the case of *S. mashanensis* and *S. jiuxuensis*, the shorter pelvic‐fin rays that do not reach the anus (vs. reaching or exceeding the anus).

*Sinocyclocheilus changlensis* differs from morphologically similar *S. tianeensis* by having pelvic‐fin rays that do not reach the anus (vs. reaching or beyond), pharyngeal teeth pattern 1,3,4–3,3,1 (vs. 2,3,4–4,3,2), vertebrae 4 + 35–36 (vs. 4 + 32–34), 11 outer rakers on the first gill arch (vs. 9). It is also distinguishable from *S. furcodorsalis* by the proximity of the posterior operculum margin to the pectoral‐fin base (vs. a more distant margin), greater lateral line scales count (42–46 vs. 37–39), pharyngeal teeth pattern 1,3,4–3,3,1 (vs. 2,3,4–4,3,2), vertebrae 4 + 35–36 (vs. 4 + 32–34), 11 outer rakers on the first gill arch (vs. 9), greater preanal length (71.5%–77.2% SL vs. 68.3%–71.2% SL), greater prepectoral length (28.7%–33.4% SL vs. 27.2%–28.1% SL), greater prepelvic length (50.3%–54.0% SL vs. 46.3%–49.1% SL), greater head length (30.5%–33.2% SL vs. 27.9%–30.1% SL), and shorter width between posterior nostrils (4.9%–6.2% SL vs. 6.4%–7.3% SL).

Our phylogeny supports the status of a new species of *S. changlensis*. The phylogenetic relationship between *S. changlensis* and *S. microphthalmus, S. anatirostris, S. anshuiensis*, and *S. luolouensis* is not close, despite all species being part of the Hongshui River system. These species do not belong to the same phylogenetic clade (Figure 5). Although an exploratory analysis of body size changes based on linear data for 12 species did not clearly distinguish *S. changlensis* from *S. furcodorsalis* , a similar analysis conducted on a subset of eight species provided support for differentiating *S. changlensis* from the remaining seven species (Figure 2). Furthermore, results from the MANOVA confirmed significant differences among the species studied (Tables 1 and 2). Likewise, the geometric morphometric analysis demonstrated that overall body size effectively distinguished *S. changlensis* from the 11 other *Sinocyclocheilus* species (Figure 4).

While *S. changlensis* shares close evolutionary relationships with *S. brevibarbatus* , *S. jiuxuensis*, *S. altishoulderus* , *S. mashanensis*, and *S. simengensis*, its morphological similarity to *S. furcodorsalis* and *S. tianeensis* contrasts with the observed genetic distances. The genetic distance between *S. changlensis* and its closest relatives is less than 1.3%, while the distance from its morphologically similar species exceeds 1.7% in the cytochrome b gene (*cytb*). The genetic distance for the ND4 gene shows that except for *S. mashanensis*, the distance to its closest relatives (0.5% and 0.7%) is equal to or smaller than that from its morphologically similar species.

A pattern of low mtDNA‐based genetic distances, despite high levels of morphological disparity, is highlighted by Mao et al. (2022) for *Sinocyclocheilus* species. Several instances of low *p*‐distances for *Sinocyclocheilus* species pairwise comparisons for the cytochrome b (*cytb*) gene are as follows: *S. huanjiangensis* (Wu et al. 2010) and *S. brevis* (Chen and Lan 1992), 0.4%; *S. hugeibarbatus* (Li, Ran, and Chen 2003) and *S. donglanensis*, 0.6%; *S. jii* (Zhang and Dai 1992) and *S. huangtianensis* (Zhu et al. 2011), 0.7%; *S. lingyunensis* and *S. donglanensis*, 0.5%; *S. longibarbatus* (Wang and Chen 1989) and *S. gracilicaudatus* (Wang et al. 2014), 0.5%; *S. wenshanensis* (Yang et al. 2017) and *S. purpureus* (Li 1985), 0.4%; *S. xunlensis* (Lan et al. 2004) and *S. gracilicaudatus* , 0.8%. For comparisons based on the ND4 gene, the *p*‐distances were as follows: between *S. furcodorsalis* and *S. altishoulderus* , 0.5%; *S. huanjiangensis* and *S. brevis* , 0.6%; *S. jii* and *S. huangtianensis*, 0.7%; and *S. wenshanensis* and *S. purpureus* , 0.7%. Notably, *S. xunlensis* and *S. gracilicaudatus* , while morphologically distinct, exhibit a relatively low genetic distance. An extreme case is observed between *S. anophthalmus* (Chen et al. 1988) and *S. maitianheensis* (Li 1992), two sister species with a genetic distance of only 0.36%, despite significant morphological disparity (Zhao and Zhang 2009).

### 4.1 Ecological Considerations

*Sinocyclocheilus changlensis* has a restricted distribution and is currently known only from its type locality, a partially open cave near the Hongshui River in Guangxi, China. The cave environment is characterized by faint light. It contains a large pool where *S. changlensis* coexists with other cave‐dwelling species from other genera, such as *Pterocryptis anomala*, *Hongshuia megalophthalmus*, and *Schistura fasciolatus*. This isolated and specialized habitat and the associated selective regimes likely contribute to speciation.

## 5 Conclusions

The evidence from this integrative analysis, combining detailed morphological, morphometric, and molecular data, supports the species status of *S. changlensis*. Its distinct morphology, characterized by the absence of eyes, a forked horn‐like structure, and specific fin and body features, is complemented by its phylogenetic separation from closely related and morphologically similar species. The genetic divergence observed in *cytb* and ND4 genes reinforces this taxonomic placement. Hence, *S. changlensis* represents a well‐defined species within the *Sinocyclocheilus* genus, contributing to the understanding of cavefish diversity and evolutionary adaptations in this unique karstic landscape.

## Author Contributions

**Yewei Liu:** conceptualization (equal), data curation (equal), formal analysis (lead), investigation (lead), methodology (equal), resources (equal), validation (equal), visualization (equal), writing – original draft (equal), writing – review and editing (equal). **Tingru Mao:** data curation (equal), formal analysis (equal), validation (equal), writing – review and editing (equal). **Hiranya Sudasinghe:** conceptualization (equal), formal analysis (equal), methodology (equal), validation (equal), writing – original draft (equal), writing – review and editing (equal). **Jiajun Zhou:** data curation (equal), investigation (equal), validation (equal), writing – review and editing (equal). **Rongjiao Chen:** data curation (equal), investigation (equal), validation (equal), writing – review and editing (equal). **Jian Yang:** data curation (equal), investigation (equal), resources (equal), supervision (equal), validation (equal), writing – review and editing (equal). **Madhava Meegaskumbura:** conceptualization (equal), funding acquisition (lead), project administration (equal), resources (equal), supervision (lead), writing – original draft (equal), writing – review and editing (equal).

## Ethics Statement

The treatment of experimental animals in this study fully complies with the Chinese Animal Welfare Law (GB/T 358922018). All animal protocols in this study were reviewed and approved by the Research Ethics Committee of Guangxi University (# GXU2024282).

## Conflicts of Interest

The authors declare no conflicts of interest.

## Supporting information


**Supporting Information S1:** Uncorrected *p*‐distance between 63 species of the genus *Sinocyclocheilus* based on mitochondrial *cytb* sequences.

**Supporting Information S2:** Uncorrected *p*‐distance between 54 species of the genus *Sinocyclocheilus* based on mitochondrial ND4 sequences.

## Data Availability

Demultiplexed sequence data are available on NCBI through PQ505198–PQ505244.

## Appendix A

**TABLE A1:** Measurements of the adult specimens of *Sinocyclocheilus changlensis* and closely related species.

Species	Voucher	Dorsal fin	Pectoral fin	Tail fin^b^	Anal fin	Pelvic fin	SL	BD	PL	DFL	DBL	PAL	ABL	AFL	PPTL	PTBL	PTFL	PPVL	PVBL
*S. changlensis*	GXU2020000035	iii, 6	i, 13	15	iii, 5	i, 7	75.0	22.5	44.4	16.2	11.1	57.0	7.0	13.6	25.0	2.6	19.6	40.5	2.9
*S. changlensis*	GXU2020000037	iii, 6	i, 14	15	iii, 5	i, 7	77.5	23.2	44.7	14.7	10.6	59.8	6.9	14.4	25.5	3.1	20.3	41.5	3.9
*S. changlensis*	GXU2020000040	iii, 7	i, 14	16	iii, 5	i, 7	93.9	29.2	55.7	18.6	13.5	69.5	9.3	17.9	28.5	3.7	22.3	48.7	4.8
*S. changlensis*	GXU2020000041^a^	iii, 6	i, 14	16	iii, 5	i, 7	137.8	39.9	80.6	26.6	17.6	102.4	11.6	23.9	39.6	5.3	36.1	69.4	6.6
*S. changlensis*	GXU2020000046	iii, 7	i, 14	16	iii, 5	i, 7	125.9	39.7	74.0	26.9	20.6	91.4	12.3	22.0	39.0	5.7	32.6	63.4	6.6
*S. changlensis*	GXU2020000047	iii, 8	i, 15	17	iii, 5	i, 7	115.1	35.2	65.2	23.2	19.0	83.7	12.1	19.0	35.2	5.1	29.3	59.3	6.6
*S. changlensis*	GXU2020000048	iii, 7	i, 15	16	iii, 5	i, 7	125.7	39.9	76.2	25.1	20.3	92.3	11.7	22.5	39.5	5.9	31.8	63.5	6.7
*S. changlensis*	GXU2020000049	iii, 7	i, 14	16	iii, 5	i, 7	127.4	40.9	74.2	23.5	20.8	91.5	12.0	21.3	37.2	5.0	31.7	64.6	6.4
*S. changlensis*	GXU2020000050	iii, 7	i, 13	17	iii, 5	i, 7	119.7	38.4	68.4	23.4	19.2	87.7	13.4	23.5	35.1	5.7	30.4	61.2	6.1
*S. changlensis*	GXU2020000051	iii, 7	i, 15	16	iii, 5	i, 7	103.3	30.4	60.4	19.7	14.9	75.4	9.7	17.0	30.9	4.1	24.1	54.7	5.1
*S. changlensis*	GXU2020000052	iii, 6	i, 14	16	iii, 5	i, 7	93.7	30.0	56.1	17.8	13.2	68.0	10.1	16.2	27.9	4.3	23.7	47.6	4.7
*S. changlensis*	GXU2020000053	iii, 6	i, 14	16	iii, 5	i, 7	117.8	39.4	68.7	23.6	16.0	89.7	10.4	21.0	35.6	5.1	30.8	61.8	5.0
*S. changlensis*	GXU2020000054	iii, 6	i, 14	16	iii, 5	i, 7	73.9	21.5	43.5	15.8	12.3	52.9	6.8	13.0	24.0	3.0	18.0	37.4	3.9
*S. changlensis*	GXU2020000055	iii, 6	i, 13	16	iii, 5	i, 7	74.3	20.6	44.4	16.5	10.6	55.1	7.5	13.8	24.5	3.5	19.9	38.6	4.1
*S. furcodorsalis*	GXU2020000010	iii, 7	i, 15	14	iii‐5	i, 7	65.5	22.4	36.4	14.7	11.5	44.7	7.5	12.7	18.3	2.8	15.6	31.8	3.9
*S. furcodorsalis*	GXU2020000011	iii, 7	i, 15	16	iii‐5	i, 7	69.1	20.1	37.3	14.2	11.5	47.5	7.5	11.4	18.8	3.0	14.1	32.5	4.0
*S. furcodorsalis*	GXU2020000012	iii, 7	i, 15	17	iii‐5	i, 7	79.0	22.5	42.4	16.3	12.4	54.2	8.1	13.6	21.6	3.2	18.0	36.6	4.4
*S. furcodorsalis*	GXU2020000013	iii, 7	i, 14	17	iii‐5	i, 7	76.3	22.5	41.3	15.3	11.8	53.7	8.5	13.3	20.9	3.5	16.5	36.4	4.1
*S. furcodorsalis*	GXU2020000014	iii, 7	i, 15	16	iii‐5	i, 7	68.4	22.2	38.7	13.2	10.1	48.7	6.7	11.7	19.2	3.4	15.0	33.6	3.6
*S. furcodorsalis*	GXU2020000058	iii, 7	i, 14	16	iii‐5	i, 8	73.0	22.5	40.1	14.8	11.6	50.4	7.9	12.9	20.2	3.6	15.0	35.2	4.1
*S. furcodorsalis*	GXU2020000059	iii, 7	i, 15	16	iii‐5	i, 7	83.8	26.8	45.4	15.6	12.8	59.2	8.8	12.8	23.3	3.1	19.3	40.1	4.9
*S. tianeensis*	GXU2020000015	iii, 7	i, 15	17	iii‐5	i, 7	83.8	26.9	47.6	18.2	13.6	57.7	8.4	14.9	24.2	4.6	21.3	41.8	5.3
*S. tianeensis*	GXU2020000016	iii, 8	i, 16	16	iii‐5	i, 7	105.0	33.1	59.4	20.0	15.6	72.5	11.1	17.2	29.3	5.0	24.8	50.6	6.2
*S. tianeensis*	GXU2020000017	iii, 7	i, 14	17	iii‐5	i, 7	97.9	32.7	56.1	19.2	15.7	70.3	9.3	16.6	28.0	4.8	23.0	49.8	5.3
*S. tianeensis*	GXU2020000018	iii, 7	i, 16	17	iii‐5	i, 7	88.8	27.1	50.7	18.2	14.3	64.2	9.2	16.5	25.9	4.0	22.5	43.9	5.4
*S. tianeensis*	GXU2020000019	iii, 7	i, 14	17	iii‐5	i, 7	84.8	27.5	49.2	18.5	14.4	60.1	8.6	16.3	25.4	3.9	21.3	43.7	4.8
*S. tianeensis*	GXU2020000056	iii, 8	i, 15	16	iii‐5	i, 7	112.6	40.6	65.7	22.2	17.4	79.3	10.6	17.2	32.0	5.4	28.0	57.8	6.3
*S. tianeensis*	GXU2020000057	iii, 7	i, 15	16	iii‐5	i, 7	106.7	36.1	61.7	20.5	17.0	74.1	10.8	17.2	31.5	4.9	27.8	53.9	6.5
*S. mashanensis*	GXU2020000001	iii, 7	i, 14	17	iii‐5	i, 7	134.4	38.3	76.9	26.7	18.7	95.2	12.2	22.7	41.1	6.0	30.4	64.59	7.4
*S. mashanensis*	GXU2020000002	iii, 7	i, 14	16	iii‐5	i, 8	151.8	47.8	90.7	30.9	23.6	105.1	14.7	28.3	44.3	6.3	40.4	72.27	9.4
*S. mashanensis*	GXU2020000003	iii, 7	i, 14	16	iii‐5	i, 8	132.9	38.3	74.8	27.1	20.2	93.8	11.9	24.5	39.5	6.1	28.8	61.14	7.8
*S. mashanensis*	GXU2020000004	iii, 8	i, 14	17	iii‐5	i, 7	113.8	36.6	65.5	28.1	20.6	80.1	11.8	22.7	35.4	5.6	31.2	54.16	6.9
*S. altishoulderus*	GXU2020000020	iii, 7	i, 14	17	iii‐5	i, 7	135.9	39.2	78.3	26.6	21.2	96.8	12.5	20.5	38.3	6.1	31.2	61.58	7.0
*S. altishoulderus*	GXU2020000021	iii, 7	i, 14	17	iii‐5	i, 7	129.3	40.0	72.8	24.8	19.8	91.1	12.7	19.3	33.3	5.3	30.4	58.06	6.8
*S. altishoulderus*	GXU2020000022	iii, 7	i, 14	17	iii‐5	i, 7	137.2	40.8	76.8	25.5	20.8	98.1	12.5	19.1	39.1	6.6	28.9	63.06	6.8
*S. brevibarbatus*	GXU2020000028	iii, 7	i, 14	17	iii‐5	i, 7	110.6	30.8	64.0	21.8	15.0	76.2	9.3	18.8	34.9	4.5	26.1	53.57	4.4
*S. brevibarbatus*	GXU2020000031	iii, 7	i, 14	17	iii‐5	i, 7	125.4	39.0	72.0	24.9	17.2	90.9	10.5	19.9	38.0	5.8	28.8	61.41	5.8
*S. brevibarbatus*	GXU2020000032	iii, 7	i, 14	17	iii‐5	i, 7	107.5	30.2	61.9	22.3	12.7	75.5	8.7	18.9	32.7	4.4	24.7	51.71	4.5
*S. brevibarbatus*	GXU2020000033	iii, 7	i, 14	17	iii‐5	i, 7	135.9	41.4	78.1	29.0	16.7	96.6	12.0	22.9	38.6	5.8	30.2	64.71	4.9
*S. brevibarbatus*	GXU2020000034	iii, 7	i, 13	17	iii‐5	i, 7	111.6	30.5	63.7	24.8	14.7	80.2	8.9	22.1	36.5	4.8	27.6	55.73	4.2
*S. jiuxuensis*	GXU2020000029	iii, 7	i, 14	17	iii‐5	i, 7	134.8	37.7	75.6	26.8	20.3	91.8	12.3	23.7	40.4	5.3	35.7	64.13	6.9
*S. jiuxuensis*	GXU2020000030	iii, 7	i, 14	17	iii‐5	i, 7	133.7	40.6	77.1	26.9	18.6	91.2	11.9	21.3	38.1	5.4	29.0	62.89	6.7
*S. flexuosdorsalis*	GXU2020000056	iii, 8	i, 13	17	iii‐5	i, 7	86.0	24.9	47.5	15.5	18.9	61.1	9.4	15.9	26.0	4.4	24.4	42.36	5.5
*S. flexuosdorsalis*	GXU2020000057	iii, 7	i, 12	17	iii‐5	i, 6	85.6	22.6	46.7	14.7	18.9	60.5	9.0	15.5	24.3	3.9	21.2	42.48	4.4
*S. flexuosdorsalis*	GXU2020000058	iii, 8	i, 13	17	iii‐5	i, 7	92.7	26.7	53.0	17.6	20.3	66.6	10.9	19.8	30.1	4.0	27.1	47.54	5.4
*S. anshuiensis*	GXU2020000068	iii, 7	i, 11	15	iii‐5	i, 7	71.8	21.1	41.7	9.1	14.1	54.7	6.9	13.1	22.5	2.9	16.2	38.35	3.0
*S. anshuiensis*	GXU2020000069	iii, 7	i, 11	16	iii‐5	i, 7	74.8	20.2	42.5	9.8	15.6	56.6	6.5	12.8	23.6	2.2	15.6	40.82	3.2
*S. anshuiensis*	GXU2020000070	iii, 7	i, 12	16	iii‐5	i, 7	77.6	21.6	45.2	10.1	14.7	59.1	7.0	11.5	24.1	2.4	14.3	40.91	2.4
*S. anshuiensis*	GXU2020000071	iii, 7	i, 11	17	iii‐5	i, 7	87.5	12.2	46.6	11.6	16.1	64.2	7.5	13.4	27.2	3.1	15.6	44.53	3.8
*S. anshuiensis*	GXU2020000072	iii, 7	i, 11	16	iii‐5	i, 7	63.8	17.4	36.9	9.1	14.4	45.8	6.3	11.3	20.1	2.5	14.7	32.51	3.0
*S. microphthalmus*	GXU2020000063	iii, 7	i, 13	17	iii‐5	i, 7	113.7	33.2	57.1	18.7	31.7	77.3	12.4	25.2	29.8	4.6	33.9	52.25	4.7
*S. microphthalmus*	GXU2020000064	iii, 7	i, 13	17	iii‐5	i, 7	112.3	27.9	55.7	19.1	28.7	76.9	9.7	21.5	30.0	4.7	33.0	50.58	5.0
*S. microphthalmus*	GXU2020000065	iii, 8	i, 12	17	iii‐6	i, 7	108.6	27.6	54.7	17.0	28.3	75.1	10.7	21.7	28.3	4.3	30.0	49.34	4.9
*S. microphthalmus*	GXU2020000066	iii, 8	i, 13	17	iii‐6	i, 7	109.3	31.9	54.3	17.7	29.3	75.2	12.4	22.5	28.0	4.0	32.5	49.3	5.4
*S. microphthalmus*	GXU2020000067	iii, 7	i, 13	17	iii‐5	i, 7	113.2	30.1	58.9	18.5	29.9	81.3	11.4	23.9	30.7	4.5	33.4	53.15	6.5
*S. anatirostris*	GXU2020000074	iii, 8	i, 10	17	iii‐5	i, 6	66.2	13.6	36.7	9.2	17.3	48.5	7.3	13.4	23.4	2.3	16.9	36.06	2.9
*S. anatirostris*	GXU2020000075	iii, 7	i, 10	17	iii‐5	i, 6	73.1	17.8	40.7	11.0	16.9	56.3	6.9	13.3	25.9	2.9	18.9	40.39	2.9
*S. luolouensis*	GXU2020000073	iii, 7	i, 13	17	iii‐5	i, 7	106.5	23.1	59.4	12.7	21.2	76.5	9.6	19.0	32.7	4.4	22.7	53.39	106.5

Species	Voucher	PVFL	CPL	CPD	HL	HD	HW	SNL	EBD	ED	IOW	IPNW	IPONW	UJL	LJL	MW	MBL	MCBL
*S. changlensis*	GXU2020000035	12.7	14.4	9.3	24.9	12.5	10.4					4.3	4.4	6.5	4.9	4.4	8.2	7.6
*S. changlensis*	GXU2020000037	13.7	15.8	9.0	25.4	12.7	10.6					4.3	4.7	6.5	5.1	4.9	8.0	7.4
*S. changlensis*	GXU2020000040	15.9	19.5	11.8	29.2	14.0	13.1					5.3	5.4	7.5	5.8	6.1	11.6	11.2
*S. changlensis*	GXU2020000041	21.7	28.1	17.6	42.0	20.3	18.3					6.6	6.8	10.9	9.9	9.0	14.1	13.6
*S. changlensis*	GXU2020000046	20.2	24.4	16.2	40.8	19.8	20.9					6.6	7.1	10.8	10.1	8.3	15.4	12.9
*S. changlensis*	GXU2020000047	19.0	22.6	16.0	36.5	16.3	17.6					6.5	6.9	9.1	7.7	7.1	13.9	10.4
*S. changlensis*	GXU2020000048	19.2	24.1	15.9	40.1	17.5	19.6					6.8	7.1	11.0	9.3	8.4	13.8	12.6
*S. changlensis*	GXU2020000049	17.5	26.4	16.8	39.8	18.5	19.1					6.8	7.2	9.7	8.6	8.1	13.1	12.2
*S. changlensis*	GXU2020000050	17.2	23.7	15.8	37.0	17.2	17.7					6.4	6.8	9.6	7.9	8.0	13.1	11.7
*S. changlensis*	GXU2020000051	15.3	21.2	12.6	32.1	15.6	15.5					5.5	6.1	8.4	7.3	7.1	12.4	11.4
*S. changlensis*	GXU2020000052	16.2	19.8	13.1	29.2	14.9	14.7					5.4	5.8	8.1	6.7	6.8	10.8	8.6
*S. changlensis*	GXU2020000053	20.5	23.8	14.2	37.2	18.6	17.9					6.2	6.5	9.5	8.3	7.5	13.0	11.3
*S. changlensis*	GXU2020000054	12.3	15.9	9.4	24.2	12.4	10.1					4.3	4.6	5.9	5.3	5.4	8.2	7.3
*S. changlensis*	GXU2020000055	13.7	14.3	9.2	24.6	12.5	10.6					4.1	4.5	5.6	4.7	5.0	8.9	8.8
*S. furcodorsalis*	GXU2020000010	11.7	14.8	9.5	19.6	9.8	10.4					3.7	4.8	5.2	4.5	4.3	7.0	6.5
*S. furcodorsalis*	GXU2020000011	10.7	15.2	9.4	19.8	10.2	10.7					3.8	5.0	5.5	4.4	4.6	7.2	5.1
*S. furcodorsalis*	GXU2020000012	11.8	17.8	10.0	22.1	11.2	10.9					4.5	5.1	5.9	4.6	4.6	8.0	5.9
*S. furcodorsalis*	GXU2020000013	11.8	16.1	10.0	22.6	10.3	11.0					4.2	5.0	5.7	4.6	4.4	7.9	7.1
*S. furcodorsalis*	GXU2020000014	10.4	13.4	9.0	20.6	10.3	9.9					3.8	4.8	5.2	4.5	4.2	6.9	6.3
*S. furcodorsalis*	GXU2020000058	12.0	16.3	9.0	21.2	10.6	10.4					4.2	5.3	5.5	4.8	4.6	7.3	6.7
*S. furcodorsalis*	GXU2020000059	12.4	16.9	11.0	23.9	10.4	12.1					3.8	5.6	6.4	5.5	5.3	8.8	8.6
*S. tianeensis*	GXU2020000015	14.0	18.6	11.0	24.9	11.8	13.6					4.2	5.9	7.9	5.8	5.2	7.6	6.3
*S. tianeensis*	GXU2020000016	16.8	23.1	14.2	29.9	12.5	15.2					6.2	6.6	9.6	7.5	6.7	14.6	13.9
*S. tianeensis*	GXU2020000017	15.9	21.3	14.2	29.2	12.8	16.0					5.2	5.9	9.1	6.3	5.7	13.2	11.4
*S. tianeensis*	GXU2020000018	15.0	19.2	11.4	26.3	11.9	14.2					5.0	5.9	7.8	6.2	5.3	12.3	9.9
*S. tianeensis*	GXU2020000019	14.4	18.5	11.0	26.1	11.6	13.5					5.5	5.7	8.0	6.5	5.7	10.1	8.5
*S. tianeensis*	GXU2020000056	16.6	23.8	16.6	33.6	13.8	16.7					5.6	6.3	9.2	8.0	7.9	17.8	16.0
*S. tianeensis*	GXU2020000057	16.9	23.2	14.9	32.9	13.4	16.1					5.2	5.6	8.7	8.4	7.5	15.3	15.1
*S. mashanensis*	GXU2020000001	23.3	29.9	18.6	43.8	23.7	22.3	16.1	5.0	8.2	11.4	10.1	10.2	12.9	11.9	8.9	8.6	11.8
*S. mashanensis*	GXU2020000002	26.4	37.4	20.6	48.9	24.6	25.2	16.7	5.3	9.1	12.8	8.0	10.9	13.8	11.0	13.9	11.9	14.5
*S. mashanensis*	GXU2020000003	24.6	30.1	16.5	41.7	20.9	20.9	14.2	4.5	7.4	9.0	7.2	8.7	13.2	11.4	10.2	11.5	11.9
*S. mashanensis*	GXU2020000004	23.4	24.2	15.5	36.9	17.9	18.9	12.5	4.8	8.1	10.3	6.2	8.1	11.2	9.7	10.6	8.7	9.0
*S. altishoulderus*	GXU2020000020	23.1	29.9	18.4	38.6	21.5	19.3	12.9	5.6	10.4	10.7	6.8	7.9	10.3	8.3	9.3	11.6	9.9
*S. altishoulderus*	GXU2020000021	22.2	30.1	18.2	35.4	19.7	19.3	13.1	4.9	9.7	10.6	7.3	7.6	10.1	6.7	7.7	13.3	12.5
*S. altishoulderus*	GXU2020000022	20.8	31.9	19.0	39.4	19.8	19.5	14.9	5.7	9.7	11.4	7.9	7.0	10.4	8.7	8.4	10.7	9.7
*S. brevibarbatus*	GXU2020000028	19.5	27.6	13.3	36.6	19.1	17.2	13.0	4.7	9.4	11.4	6.5	7.4	10.8	10.5	9.9	5.4	5.5
*S. brevibarbatus*	GXU2020000031	21.3	28.4	15.7	38.0	19.4	19.5	12.9	5.7	11.5	11.3	7.5	7.9	9.9	9.1	9.7	8.1	9.9
*S. brevibarbatus*	GXU2020000032	20.0	26.1	11.2	32.9	17.8	15.7	10.2	6.0	10.3	10.5	5.8	7.1	9.5	9.0	9.0	5.8	6.3
*S. brevibarbatus*	GXU2020000033	25.0	29.8	16.1	40.4	22.1	21.7	13.0	6.2	12.7	11.5	7.3	8.3	12.0	11.0	11.7	4.7	6.3
*S. brevibarbatus*	GXU2020000034	22.9	25.7	12.5	34.5	18.3	15.6	10.4	5.9	10.5	9.0	6.4	6.7	10.8	10.1	8.9	4.0	3.1
*S. jiuxuensis*	GXU2020000029	24.2	33.0	15.9	40.7	20.2	17.50	14.3	3.6	9.3	10.8	7.1	8.7	11.8	10.0	9.0	9.1	8.3
*S. jiuxuensis*	GXU2020000030	23.1	33.1	15.5	40.2	19.9	19.0	13.5	4.3	10.3	10.8	8.0	8.3	10.1	9.3	8.3	8.4	8.2
*S. flexuosdorsalis*	GXU2020000056	16.2	17.5	11.9	26.8	14.1	11.7	9.2	2.1	5.3	5.2	5.1	4.7	7.2	6.8	5.9	14.4	14.9
*S. flexuosdorsalis*	GXU2020000057	15.3	16.9	10.8	25.2	13.1	11.6	8.3	2.2	4.7	5.3	4.2	4.2	6.4	5.7	4.9	9.6	11.0
*S. flexuosdorsalis*	GXU2020000058	18.9	18.9	12.1	28.9	14.5	12.8	9.7	3.0	6.3	5.9	5.1	5.0	8.2	6.3	6.0	14.4	15.8
*S. anshuiensis*	GXU2020000068	11.0	13.2	8.1	22.2	13.0	9.3					3.3	4.1	5.3	4.6	3.5	7.0	8.1
*S. anshuiensis*	GXU2020000069	12.0	14.6	8.4	23.4	14.4	8.8					3.0	3.9	4.9	4.1	3.5	8.9	8.0
*S. anshuiensis*	GXU2020000070	10.7	14.9	8.6	23.6	15.6	9.7					3.4	4.0	5.2	5.0	3.7	8.6	7.3
*S. anshuiensis*	GXU2020000071	11.9	17.6	9.6	28.3	17.1	11.5					3.9	5.1	6.2	5.9	4.8	9.0	7.9
*S. anshuiensis*	GXU2020000072	10.5	12.0	8.1	20.8	12.7	8.5					3.1	3.9	5.0	4.7	3.1	6.5	5.6
*S. microphthalmus*	GXU2020000063	25.6	25.3	12.3	28.9	16.8	14.8	9.0	1.6	4.9	8.4	4.8	7.8	6.2	5.8	6.2	13.3	11.5
*S. microphthalmus*	GXU2020000064	24.6	26.5	10.8	28.0	15.3	14.0	8.9	1.4	5.1	8.1	5.0	6.0	7.4	5.7	5.9	10.3	8.9
*S. microphthalmus*	GXU2020000065	23.7	23.9	10.8	27.1	14.8	13.2	8.2	1.4	4.7	7.3	4.8	7.5	7.0	6.1	5.4	8.7	8.8
*S. microphthalmus*	GXU2020000066	24.4	23.7	12.2	27.5	14.5	13.8	9.3	1.5	4.9	7.5	5.0	6.1	6.4	5.8	5.4	9.2	8.9
*S. microphthalmus*	GXU2020000067	26.2	26.3	11.6	29.6	16.5	15.0	9.5	1.5	4.9	8.5	4.8	7.0	7.0	6.2	6.0	13.2	12.7
*S. anatirostris*	GXU2020000074	14.7	13.0	5.2	21.7	12.7	8.9					3.5	4.4	5.7	4.9	4.1	6.6	5.0
*S. anatirostris*	GXU2020000075	14.1	15.7	6.8	25.4	15.1	11.1					3.8	5.2	7.1	6.0	5.2	6.5	5.2
*S. luolouensis*	GXU2020000073	19.0	22.1	10.2	31.0	17.0	14.2	9.4	2.2	6.0	8.0	5.4	6.0	7.7	7.3	6.2	7.6	9.5

*Note:* All units in mm.

^a^ For the holotype.

^b^ Branched rays.

**TABLE A2:** Voucher information, and GenBank numbers for all samples used.

Taxon	Cytb voucher number	ND4 voucher number	Accession No.		Source
			Cyt b	ND4	
*S. altishoulderus*_Liu041	Liu041	Liu041	**PQ505198**	**PQ505222**	This study
*S. altishoulderus*_Liu042	Liu042	Liu042	**PQ505199**	**PQ505223**	This study
*S. altishoulderus*_Liu043	Liu043	Liu043	**PQ505200**	**PQ505224**	This study
*S. altishoulderus*_Liu044	Liu044	—	**PQ505201**	—	This study
*S. altishoulderus*_Liu045	Liu045	Liu045	**PQ505202**	**PQ505225**	This study
*S. anatirostris*_GZNU_YZ01	GZNU_YZ01	GZNU_YZ01	NC069226	NC069226	NCBI
*S. angularis*_GZNU20180420001	GZNU20180420001	GZNU20180420001	MZ636514	MZ636514	NCBI
*S. angularis*_GZNU202001332	GZNU202001332	GZNU202001332	MW362289	MW362289	NCBI
*S. angustiporus*_XH1203	XH1203	XH1203	AY854702	AY854759	NCBI
*S. anophthalmus*_XH3001	XH3001	XH3001	AY854715	AY854772	NCBI
*S. anophthalmus*_XH3002	XH3002	XH3002	AY854716	AY854773	NCBI
*S. anshuiensis*_NC027169	—	—	NC027169	NC027169	NCBI
*S. aquihornes*_S28	S28	S28	PQ155086	PQ155094	NCBI
*S. bicornutus*_NC031382	—	—	NC031382	NC031382	NCBI
*S. bicornutus*_XH8301	XH8301	XH8301	AY854730	AY854787	NCBI
*S. bicornutus*_XH8302	XH8302	XH8302	AY854731	AY854788	NCBI
*S. bicornutus*_XH8303	XH8303	XH8303	AY854732	AY854789	NCBI
*S. brevibarbatus*_1	GX0064‐L20‐13	GX0066	MT373106	MW548423	NCBI
*S. brevis*_1	GX0155	LIU108	MT373105	MW548424	NCBI
*S. changlensis*_LM004	LM004	LM004	**PQ505203**	**PQ505226**	This study
*S. changlensis*_LM110	LM110	LM110	**PQ505204**	**PQ505227**	This study
*S. changlensis*_LM111	LM111	LM111	**PQ505205**	**PQ505228**	This study
*S. changlensis*_LM112	LM112	LM112	**PQ505206**	**PQ505229**	This study
*S. changlensis*_Liu198	Liu198	Liu198	**PQ505207**	**PQ505230**	This study
*S. cyphotergous*_GZNU20150811002	GZNU20150811002	GZNU20150811002	MW024370	MW024370	NCBI
*S. cyphotergous*_NC072977	—	—	NC072977	NC072977	NCBI
*S. cyphotergous*_XH2701	XH2701	XH2701	AY854711	AY854768	NCBI
*S. donglanensis*_1	CA141	LIU050	AB196441	MW548425	NCBI
*S. flexuosdorsalis*_S66	S66	—	OQ718397	—	NCBI
*S. furcodorsalis*_GX0185	GX0185	GX0185	**PQ505208**	**PQ505231**	This study
*S. furcodorsalis*_LIU005	LIU005	LIU005	**PQ505209**	**PQ505232**	This study
*S. furcodorsalis*_LIU006	LIU006	LIU006	**PQ505210**	**PQ505233**	This study
*S. furcodorsalis*_LIU007	LIU007	LIU007	**PQ505211**	**PQ505234**	This study
*S. furcodorsalis*_XH2202	XH2202	XH2202	AY854709	AY854766	NCBI
*S. gracilicaudatus*_S67	S67	—	OQ718398	—	NCBI
*S. grahami*_NC013189	—	—	NC013189	NC013189	NCBI
*S. grahami*_XH0701	XH0701	XH0701	AY854694	AY854751	NCBI
*S. grahami*_XH4404	XH4404	XH4404	AY854696	AY854753	NCBI
*S. guanyangensis*_1	GX0173	LIU016	MT373108	MW548426	NCBI
*S. guilinensis*_1	GX0073	LIU057	MT373104	MW548427	NCBI
*S. guishanensis*_XH5401	XH5401	XH5401	AY854722	AY854779	NCBI
*S. guiyang*_IHB_202012250001	IHB_202012250001	IHB_202012250001	OR141734	—	NCBI
*S. huangtianensis*_1	GX0175	LIU129	MT373109		NCBI
*S. huaningensis*_XH3701	XH3701	XH3701	AY854718	AY854775	NCBI
*S. huanjiangensis*_1	GX0124	LIU087	MT373103	MW548429	NCBI
*S. hugeibarbus*_MW014319	—	—	MW014319	MW014319	NCBI
*S. huizeensis*_NC044072	—	—	NC044072	NC044072	NCBI
*S. huizeensis*_OK505604	—	—	OK505604	OK505604	NCBI
*S. hyalinus*_XH4701	XH4701	XH4701	AY854721	AY854778	NCBI
*S. jii*_XH8101	XH8101	XH8101	AY854727	AY854784	NCBI
*S. jiuxuensis*_Liu425	Liu425	Liu425	**PQ505212**	**PQ505235**	This study
*S. jiuxuensis*_Liu426	Liu426	Liu426	**PQ505213**	**PQ505236**	This study
*S. jiuxuensis*_Liu428	Liu428	Liu428	**PQ505214**	**PQ505237**	This study
*S. jiuxuensis*_XH8501	XH8501	XH8501	AY854736	AY854793	NCBI
*S. jiuxuensis*_XH8502	XH8502	XH8502	AY854737	AY854794	NCBI
*S. lateristriatus*_XH1301	XH1301	XH1301	AY854704	AY854761	NCBI
*S. lateristriatus*_XH1302	XH1302	XH1302	AY854705	AY854762	NCBI
*S. lateristriatus*_XH1601	XH1601	XH1601	AY854707	AY854764	NCBI
*S. lingyunensis*_NC056143	—	—	NC056143	NC056143	NCBI
*S. lingyunensis*_XH0502	XH0502	XH0502	AY854691	AY854748	NCBI
*S. lingyunensis*_XH2001	XH2001	XH2001	AY854692	AY854749	NCBI
*S. lingyunensis*_XH3301	XH3301	XH3301	AY854693	AY854750	NCBI
*S. longibarbatus*_GZNU2019102022	GZNU2019102022	GZNU2019102022	MW024371	MW024371	NCBI
*S. longibarbatus*_MT361975	—	—	MT361975	MT361975	NCBI
*S. longibarbatus*_NC056194	—	—	NC056194	NC056194	NCBI
*S. longicornus*_PZ01	PZ01	—	MZ634123	MZ634125	NCBI
*S. longicornus*_PZ02	PZ02	—	MZ634124	MZ634126	NCBI
*S. longshanensis_*S22	S22	S22	PQ155085	PQ155093	NCBI
*S. macrocephalus*_XH0103	XH0103	XH0103	AY854683	AY854740	NCBI
*S. macrocephalus*_XH0110	XH0110	XH0110	AY854684	AY854741	NCBI
*S. macrolepis*_XH8201	XH8201	XH8201	AY854729	AY854786	NCBI
*S. macrophthalmus*_XH8401	XH8401	XH8401	AY854733	AY854790	NCBI
*S. macrophthalmus*_XH8402	XH8402	XH8402	AY854734	AY854791	NCBI
*S. macrophthalmus*_XH8403	XH8403	XH8403	AY854735	AY854792	NCBI
*S. maculatus*_T12	T12	—	MF325008	—	NCBI
*S. maitianheensis*_XH2301	XH2301	XH2301	AY854710	AY854767	NCBI
*S. malacopterus*_XH0901	XH0901	XH0901	AY854697	AY854754	NCBI
*S. malacopterus*_XH0902	XH0902	XH0902	AY854698	AY854755	NCBI
*S. malacopterus*_XH2501	XH2501	XH2501	AY854699	AY854756	NCBI
*S. malacopterus*_XH5501	XH5501	XH5501	AY854700	AY854757	NCBI
*S. mashanensis*_1	GX0026	LIU066	MT373107	MW548430	NCBI
*S. mashanensis*_LYW014	LYW014	LYW014	**PQ505215**	**PQ505238**	This study
*S. mashanensis*_LYW015	LYW015	LYW015	**PQ505216**	**PQ505239**	This study
*S. microphthalmus*_MN145877	—	—	MN145877	MN145877	NCBI
*S. microphthalmus*_XH0402	XH0402	XH0402	AY854687	AY854744	NCBI
*S. microphthalmus*_XH1801	XH1801	XH1801	AY854688	AY854745	NCBI
*S. microphthalmus*_XH2101	XH2101	XH2101	AY854689	AY854746	NCBI
*S. microphthalmus*_XH2102	XH2102	XH2102	AY854690	AY854747	NCBI
*S. multipunctatus*_XH2801	XH2801	XH2801	AY854712	AY854769	NCBI
*S. oxycephalus*_MW548263	—	—	MW548263	MW548263	NCBI
*S. oxycephalus*_XH0201	XH0201	XH0201	AY854685	AY854742	NCBI
*S. punctatus*_MK610346	—	—	MK610346	MK610346	NCBI
*S. punctatus*_MW014318	—	—	MW014318	MW014318	NCBI
*S. punctatus*_NC058003	—	—	NC058003	NC058003	NCBI
*S. purpureus*_NC063103	—	—	NC063103	NC063103	NCBI
*S. qiubeiensis*_WD13	WD13	—	MF324996	—	NCBI
*S. qujingensis*_NC043910	—	—	NC043910	NC043910	NCBI
*S. rhinocerous*_NC027168	—	—	NC027168	NC027168	NCBI
*S. rhinocerous*_XH3901	XH3901	XH3901	AY854720	AY854777	NCBI
*S. ronganensis*_NC032385	—	—	NC032385	NC032385	NCBI
*S. sanxiaensis*_OP745534	—	—	OP745534	OP745534	NCBI
*S. simengensis*_S75	S75	—	OQ718406	—	NCBI
*S. tianeensis*_GX0182	GX0182	GX0182	**PQ505217**	**PQ505240**	This study
*S. tianeensis*_GX0183	GX0183	GX0183	**PQ505218**	**PQ505241**	This study
*S. tianeensis*_LIU001	LIU001	LIU001	**PQ505219**	**PQ505242**	This study
*S. tianeensis*_LIU002	LIU002	LIU002	**PQ505220**	**PQ505243**	This study
*S. tianeensis*_LIU003	LIU003	LIU003	**PQ505221**	**PQ505244**	This study
*S. tianlinensis*_1	GX0087	GX0121	MT373102	MW548431	NCBI
*S. tingi*_XH1001	XH1001	XH1001	AY854701	AY854758	NCBI
*S. tingi*_YNUST201406180002	YNUST201406180002	YNUST201406180002	NC039594	NC039594	NCBI
*S. wenshanensis*_MW553076	—	—	MW553076	MW553076	NCBI
*S. wenshanensis*_YNUSW20160703016	YNUSW20160703016	YNUSW20160703016	NC_060737	NC_060737	NCBI
*S. wumengshanensis*_NC039769	—	—	NC039769	NC039769	NCBI
*S. xichouensis*_S78	S78	—	OQ718409	—	NCBI
*S. xiejiahuai_*S46	S46	S46	PQ165088	PQ165088	NCBI
*S. xingyiensis*_GZNUSLS202008177	GZNUSLS202008177	—	ON573218	—	NCBI
*S. xunlensis*_IHBCY04050268	IHBCY04050268	IHBCY04050268	HM536791	HM536710	NCBI
*S. xunlensis*_IHB_04050268	IHB_04050268	IHB_04050268	EU366187	EU366184	NCBI
*S. xunlensis*_IHB_04050270	IHB_04050270	IHB_04050270	EU366190	EU366185	NCBI
*S. yangzongensis*_XH6101	XH6101	XH6101	AY854725	AY854782	NCBI
*S. yangzongensis*_XH6102	XH6102	XH6102	AY854726	AY854783	NCBI
*S. yimenensis*_IHB_2006645	IHB_2006645	IHB_2006645	EU366192	EU366179	NCBI
*S. yimenensis*_IHB_2006646	IHB_2006646	IHB_2006646	EU366191	EU366180	NCBI
*S. yishanensis*_1	GX0070	LIU095	MT373101	MW548432	NCBI
*S. yishanensis*_MK387704	—	—	MK387704	MK387704	NCBI
*S. zhenfengensis*_1	—	—	MK610342	MK610347	NCBI
*S. zhenfengensis*_MW014317	—	—	MW014317	MW014317	NCBI
*Barbodes laticeps*_XH9001	XH9001	XH9001	AY854738	AY854795	NCBI
*Barbodes laticeps*_XH9002	XH9002	XH9002	AY854739	AY854796	NCBI
*Cyprinus carpio*_OL840285	qingtian1	qingtian1	OL840285	OL840285	NCBI

*Note:* —, not available. The bold values in this study indicate that these sequences are newly sequenced.
